# High-Yield Prebiotic Polymerization of 2′,3′-Cyclic Nucleotides under Wet–Dry Cycling

**DOI:** 10.1021/acscentsci.5c00488

**Published:** 2025-06-28

**Authors:** Federico Caimi, Juliette Langlais, Francesco Fontana, Sreekar Wunnava, Tommaso Bellini, Dieter Braun, Tommaso P. Fraccia

**Affiliations:** † Department of Medical Biotechnologies and Translational Medicine, 9304 University of Milan, via Fratelli Cervi 93, I-20090 Segrate, Milano, Italy; ‡ Systems Biophysics, 9183 Ludwig-Maximilians-University Munich, Amalienstr. 54, 80799 Munich, Germany; § Department of Pharmacological and Biomolecular Sciences, University of Milan, via Balzaretti 9, I-20133 Milano, Italy

## Abstract

The spontaneous formation of RNA polymers is a fundamental yet challenging step for the origin of life. Here we show that 2′,3′-cyclic nucleotides of all four nucleobases efficiently polymerize without external activators when subjected to wet–dry cycling at room temperature in a mild alkaline pH range. We found conditions where oligomerization yields (Y) are enhanced by wet–dry cycling, reaching Y ≈ 70% for guanosine and Y ≥ 20% for other nucleobases. Microscopy monitoring during the drying process indicates that guanosine’s higher reactivity stems from its self-assembly propensity at pH ≤ 10. At pH 11, guanosine ordering is disfavored, leading to a nearly stoichiometrically balanced polymerization of the four nucleotides with Y = 36%. Only water is added at each cycle, mimicking humid nights and dry days on early Earth. This leads to a broad distribution of A, U, G, and C mixed sequence oligomers, up to 6% of 4-mer and 0.1% of 10-mer, paving the way for RNA replication and evolution through subsequent templated ligation under the same pH. The combination of simple boundary conditions and a pathway toward RNA evolution makes this process a compelling model for the prebiotic origin of RNA on early Earth.

## Graphical abstract

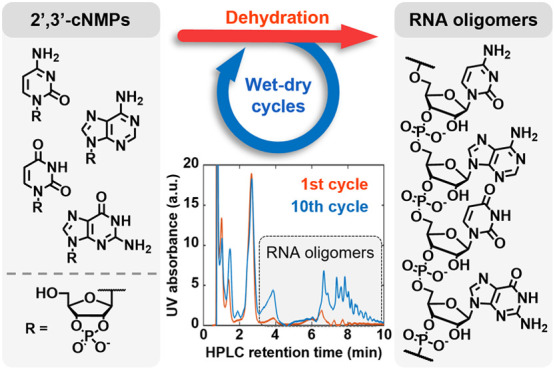

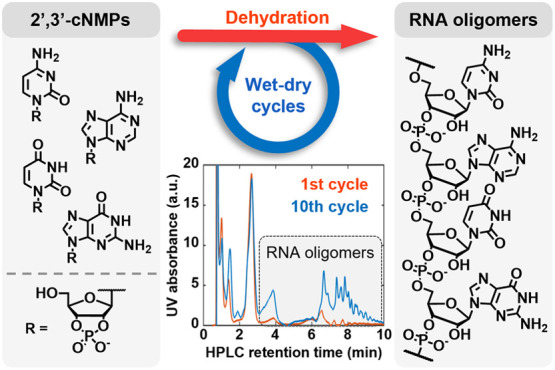

## Introduction

It is widely accepted that RNA is a fundamental molecule for the origin of life.^1,2^ However, the prebiotic emergence of RNA is still an unsolved question.^3^ Polymerization from single nucleotides poses significant challenges, due to the inefficiency in water and the predominance of hydrolysis over condensation.^4,5^ The use of activated nucleotides, such as 5′-phosphorimidazolides, on the surface of clays, such as montmorillonite,^6^ or the presence of prebiotically improbable ions, such as Pb^2+^ and Zn^2+^,^7,8^ partially mitigated the problem. Only 5′-phosphor­imidazolides of adenosine (5′-ImpA) have been reported to polymerize efficiently enough, with a maximum conversion yield of 61% in the presence of montmorillonite after 3 days (10 nt max length),^6^ or 25% in the presence of Zn^2+^ after 10 days (4 nt max length),^7^ or 50% in the presence of Pb^2+^ after 7 days (5 nt max length).^8^ While the availability of such activated nucleotides on early Earth is yet to be demonstrated, a more intriguing alternative is provided by 2′,3′-cyclic phosphate nucleotides (cNMPs) (Figure 1a) since prebiotic phosphorylation of nucleosides leading to 2′,3′-cNMPs has been shown to be plausible^9–11^ and because cyclic 2′,3′-phosphate is the natural product of RNA phosphodiester bonds cleavage.^12^

**Figure 1:**
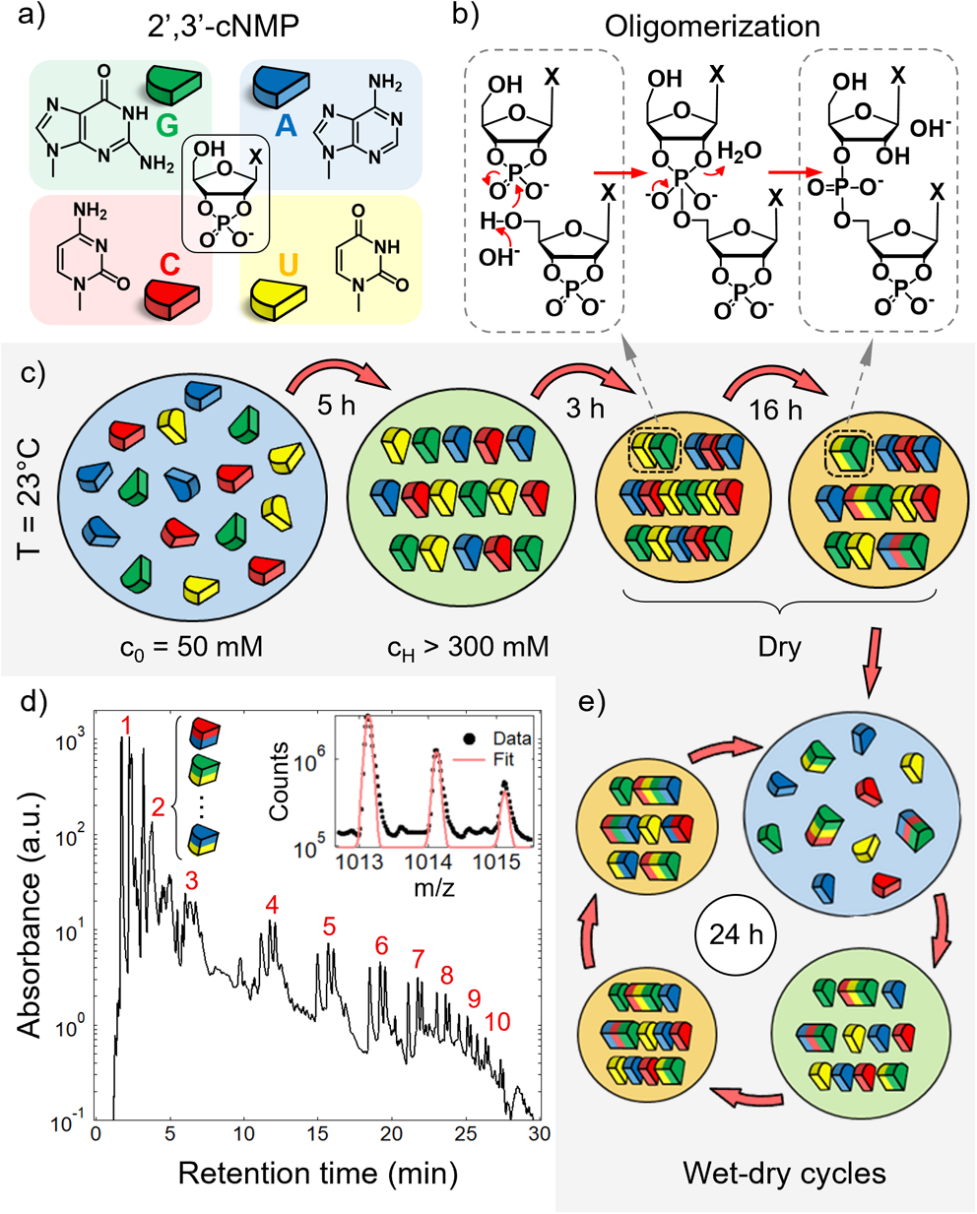
Polymerization by wet–dry cycling of 2′,3′-cyclic phosphate nucleotides. a) Molecular structures and sketches of the four canonical 2′,3′-cyclic phosphate nucleotides (2′,3′-cNMPs) studied in this work. b) Reaction scheme of the base-catalyzed transesterification leading to the formation of phosphodiester bonds of RNA oligomers. c) Schematic illustration of the evaporation process of an aqueous solution (blue) containing a 2′,3′-cNMP mixture of all four different nucleotides at room temperature. During drying, depending on pH and nucleotide species, the solution may enter a regime of supramolecular organization at sufficiently large concentration, c_H_ (green). In a fully dry condition (orange), oligomerization proceeds by transesterification as depicted in panel b. d) HPLC-MS chromatogram (log scale) of the oligomerization products in AUGC mixture (pH 10) after 10 wet–dry cycles. The groups of peaks corresponding to oligomers with the same length of ≤10 nucleotides are labeled in red. Inset: Section of the MS spectrum overlaying the isotope distribution of the polymerization product GGU-2′/3′P in the z = 1 charged state. The fit is used for the quantification of the product. e) Wet–dry cycles are implemented by new additions of pure water to the dry state with a period of t = 24 h.

The amine-catalyzed polymerization of 2′,3′-cAMP has been reported to reach 69% yield for 3 days of dry incubation at room temperature and in the presence of an excess of 1,2-diaminoethane at pH 9.5.^13,14^ In a previous work, we further demonstrated the oligomerization of all four cNMPs without catalyst or activator, under dry heating (40–80 °C) and mildly alkaline conditions; low yields were reported, with a maximum reached at pH 10: 3% at 40 °C for G and <0.1% for A, U, or C.^15^ In a follow-up study, we observed that drying at even lower temperatures (room temperature or 4 °C) improved the polymerization (15% for G and 2% for the others at room temperature). Furthermore, we reported that the polymerization proceeded by a base catalysis mechanism and that the addition of amino acids positively affected the reaction.^16^ Other strategies for RNA polymerization have been investigated in the past, including different substrate nucleotides, such as monophosphates,^17^ triphosphates,^18^ and 3′,5′-cyclic phosphates,^19–21^ and different protocols. For comparison of the results and reaction conditions, we have compiled a possibly incomplete overview of RNA nucleotide polymerization to provide more details of previous experimental efforts on this question (Table 1).

**Table 1:** Comparison with Other Nucleotide Polymerization Strategies Reported in the Literature^a^

Article	Nucleotides	Added reactants	Best reaction conditions	Max yield	Max length detected (nt)	Analysis methods	Notes
Current work	2′,3′-cGMP, 2′,3′-cCMP, 2′,3′-cAMP, 2′,3′-cUMP sodium salt	KOH	Dry phase, pH 10–12,	65% (G), 19% (U),	10 (G), 4 (U),	HPLC-UV,	Slow drying (*t* = 8–10 h) without any gas flow or vacuum; heteropolymers were reported
			T = 23 °C, t = 24 h	17% (A), 28% (C)	4 (A), 5 (C)	HPLC-MS	
		KOH	5–10 dry–wet cycles, initial pH 10–11,	70% (G), 29% (U),	10 (G), 6 (U),	HPLC-UV,	Only water addition for each cycle; heteropolymers were reported
			T = 23 °C, t = 24 h/cycle	31% (A), 29% (C)	6 (A), 6 (C)	HPLC-MS	
Rout et al. 2024 ^16^		KOH	Dry phase, pH 10–12,	16% (G), 1% (U),	10 (G), 4 (U),	HPLC-UV,	Rapid drying (*t* < 30 min) with nitrogen flow; heteropolymers were reported
			room T, t = 20 h	2% (A), 1.5% (C)	3 (A), 4 (C)	HPLC-MS	
		KOH, 50 mM valine	Dry phase, pH 10–12,	35% (G), 7% (U),	10 (G), 6 (U),	HPLC-UV,	
			room T, t = 20 h	7.5% (A), 7% (C)	6 (A), 7 (C)	HPLC-MS	
Dass et al. 2022 ^15^		KOH, NaOH	Dry phase, pH 10,	3% (G), 0.1% (U),	10 (G), 5 (U),	HPLC-UV,	Drying under ambient conditions; heteropolymers were reported
			T = 40 °C, t = 18 h	0.01% (A), 0.003% (C)	4 (A), 2 (C)	HPLC-MS	

Verlander et al. 1973, Verlander et al. 1974 ^13,14^	2′,3′-cAMP ammonium salt	1,2-Diaminoethane, NH_2_H_2_PO_4_, urea, H_3_PO_4_, NH_3_	Dry phase, pH 9.5,	68.6–79.1%	6–13	^33^P-labeled cAMP, paper chromat./electroph., gel chromatography	Vacuum or ambient conditions; high yields with diaminoethane or diaminopropane
			T = 24 °C, t = 3–40 days				

Jerome et al. 2022 ^18^	GTP, CTP, ATP, UTP	Glass surfaces	Dry phase, pH 7.5,	Not measured/provided	14 and higher MW products	[α-^32^P]-NMP incorporation/PAGE ultrafiltration	No clear identification of higher MW products; heteropolymers were investigated
			T = 25 °C, t = 20–144 h				

Costanzo et al. 2009 ^19^	3′,5′-cAMP, 3′,5′-cGMP	Tris-HCl	Liquid phase, pH 8.2,	Not measured/provided	35 (G), 4–8 (A), higher MW products	[γ-^32^P]-labeling and PAGE	No subsequent replication of the results from other groups
			T = 85 °C, t = 0.5–200 h				

Morasch et al. 2014 ^20^	3′,5′-cGMP “never dry” synthesis	—	Dry phase,	2%	40	SYBR-gold PAGE, MALDI-TOF	Vacuum dried; no polymerization in ref 19 conditions
			T = 50 °C, t = 15 h				
Wunnava et al. 2021 ^21^		NaCl	Dry phase, pH 3,	0.01%	12	SYBR-gold PAGE, HPLC-MS	Vacuum dried
			T = 80 °C, t = 20 h				

Ferris and Ertem 1992 ^6^	5′-ImpA	Montmorillonite, NaCl, MgCl_2_	Liquid phase, pH 8,	61%	10	HPLC-UV	Liquid conditions on washed clay; heteropolymers were reported
			room T, t = 72 h				

Rajamani et al. 2008 ^17^	AMP	POPC, POPA, LPC rehydration in 1 mM HCl	5–7 dry–wet cycles, pH: initial 6.8, final 2.2,	3% (A)	100 (PAGE), 50 (Nanopore), 10 (HPLC)	[γ-^32^P]-labeling PAGE, HPLC, HPLC-MS, Nanopore sequence	Synthesis RNA-like polymers due to loss of purines at low pH
			T = 90 °C, t = 30–120 min/cycle				

Dagar et al. 2020 ^31^	2′,3′-cAMP,	POPC	10–30 dry–wet cycles, pH 8.5,	11.5% (2′,3′-cA),	4 (2′,3′-cA),	HPLC-UV, TOF-MS	Carbon dioxide flow during drying; pure water and panamic spring water
	2′,3′-cCMP,		T = 90 °C, t = 24 h/cycle	21% (2′,3′-cC),	3 (2′,3′-cC),		
	3′,5′-cAMP,			0.3% (3′,5′-cA),	2 (3′,5′-cA),		
	3′,5′-cCMP			14.5% (3′,5′-cC)	2 (3′,5′-cC)		

a Where not directly provided, yields data have been calculated from graphs or tables from the relative references and may contain approximations. Strategies dealing with EDC or carbodiimides have not been considered.

Wet–dry cycling has been shown to play a fundamental role in origin of life scenarios, driving prebiotic synthesis, polymerization by condensation, and compartment assembly.^22–30^ Recently, another group investigated the effect of wet–dry cycling (at pH 8 and 90 °C) on cyclic nucleotides and in particular 2′3′-cAMP and 2′3′-cCMP: polymeric material (<4 nt long products detected) was reported to increase with subsequent cycles, although this was limited by competition with hydrolysis.^31^ Here we lowered the temperature but increased the pH. We systematically investigated wet–dry cycles at room temperature (T = 23 °C) within a pH range of 9–12. We found high-yield polymerization of all four cNMPs via dehydration–rehydration cycles (Figure 1c,e) without the addition of external activators. Compared to previously reported experiments at room temperature, where rapid drying was caused by nitrogen flow,^16^ we show here that slow evaporation without any gas flow (8 to 10 h, see Methods) significantly improved the yield and length distribution of the oligomers. Both were further improved by subsequent dehydration–rehydration cycles. This is illustrated in Figure 1d, where we show an HPLC chromatogram of the mixture of all four nucleotides after 10 cycles, revealing the emergence of distinct peaks at longer retention times corresponding to high-yield production of RNA oligomers up to at least 10 nucleotides in length. Analysis by HPLC-MS allowed detection and quantification of each distinct product, accounting for the compositional diversity of the resulting RNA oligomers (Figure 1d, inset, and SI Methods).

All experiments reported here started with 50 mM reactants and unstructured, isotropic, aqueous solutions (blue shading in the sketches of Figure 1c,e and Methods). Cycles were produced by adding pure water (no ions or buffers), a choice meant to mimic the behavior of ponds subjected to evaporation and rehydration processes by natural cycles such as day–night, rains, and tidal and seasonal successions. During the slow dehydration, as the concentration of cNMPs increases, some of the samples, the ones containing G, become ordered either as high-concentration aqueous solutions (green shading) or in a dried state (orange shading). Guanosines are expected to self-assemble in columns of planar hydrogen-bonded guanosine quartets (G-quartets) that can in turn collectively organize into liquid crystal and crystalline structures.^32–34^ Such forms of anisotropic molecular ordering are readily identified by polarized transmitted optical microscopy (PTOM). Indeed, the collective alignment on the micrometer length scale of the purine plates provided by liquid crystal ordering of G-quartets gives rise to a local birefringence that is large enough for detection. We thus routinely performed PTOM to characterize the samples during the wet–dry cycles. This analysis enabled us to evaluate the interplay between physical molecular ordering and the chemical polymerization process.

## Results and Discussion

### Polymerization of 2′,3′-Cyclic NMPs at Mildly Alkaline pH in Single Drying Processes

Oligomerization experiments were performed on solutions comprising either single 2′,3′-cNMP (N = A, U, G, C) species, binary Watson–Crick pairs (AU and GC), or mixtures of all four nucleotides (AUGC). Aqueous solutions with initial total cNMP concentration c_0_ = 50 mM were evaporated over 24 h at room temperature. Potassium hydroxide (KOH) was used to set the initial pH at values ranging from 6 to 12, unless specified otherwise (Methods). The HPCL, HPLC-MS, and PTOM results we obtained from a single drying process are summarized in Figure 2.

**Figure 2:**
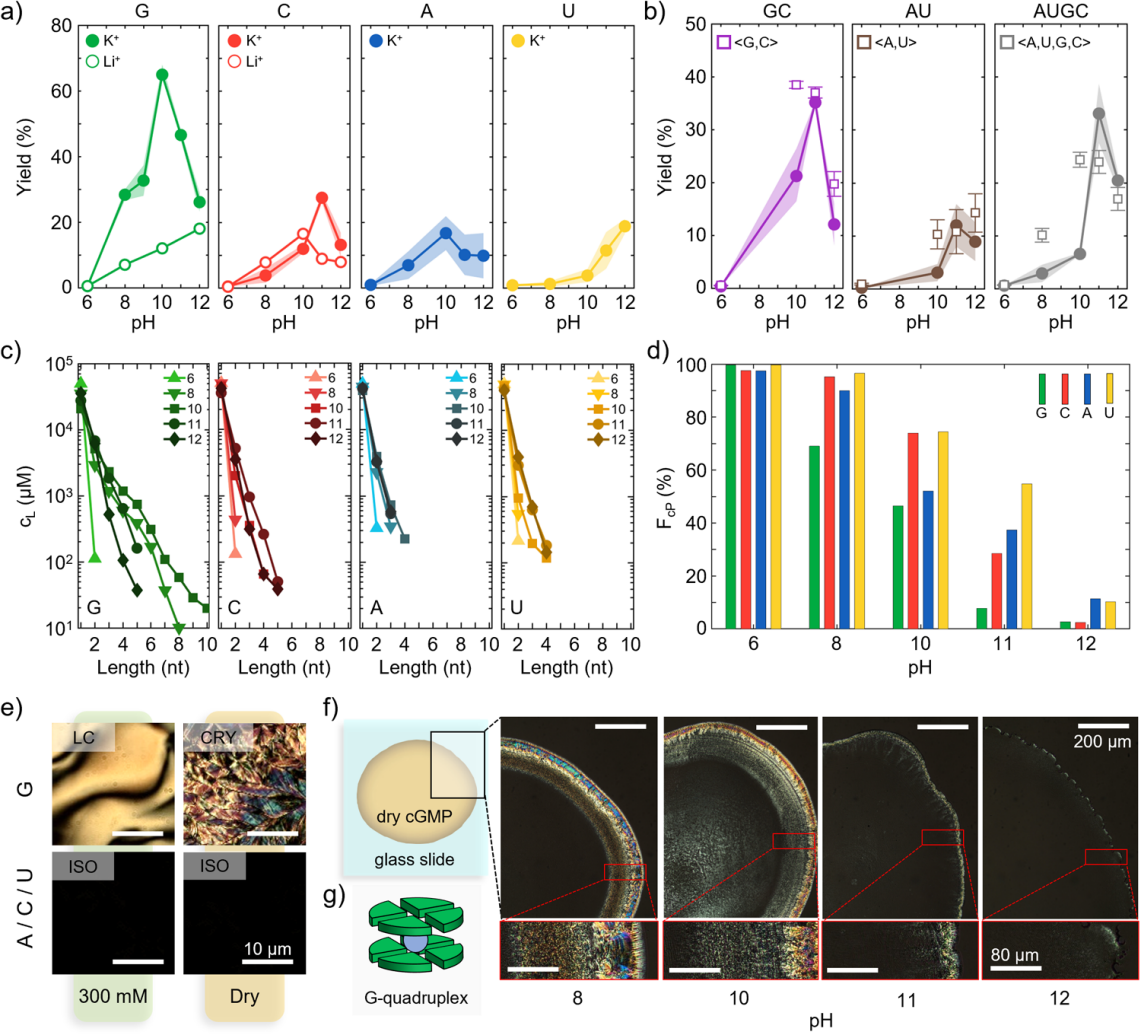
Polymerization of 2′,3′-cNMP after a single dehydration at mildly alkaline pH. a,b) Polymerization yields as a function of the initial pH for solutions of (a) individual 2′,3′-cNMP species and (b) binary and quaternary mixtures after 24 h drying at room temperature at a total concentration of 50 mM. Data are obtained by averaging HPLC and HPLC-MS measurement, and colored shading marks the confidence interval (SI Methods). c) Length distribution of the formed RNA oligomers as a function of pH. d) Fraction of cyclic terminal phosphates, F_cP_, over the total amount of molecules after the reaction, calculated considering monomers and oligomers at the same time. e) Polarized transmitted optical microscopy (PTOM) images through crossed polarizers of 2′,3′-cNMP solutions during evaporation at pH 10, in concentrated (left) and dry (right) states, showing the formation of liquid crystal and crystal phases only in the presence of 2′,3′-cGMP. f) PTOM images through crossed polarizers of the dry state of 2′,3′-cGMP droplets showing a reduction of the birefringent crystalline domains at increasing pH. g) Sketch of G-quadruplex organization.

The oligomerization yields strongly depended on both the nucleobase and pH (Figures 2a,b and S16, S17). All nucleotides and mixtures exhibited only negligible oligomerization at pH 6, while the reaction efficiency increased at alkaline pH values. This behavior was consistent with previous observations performed at higher temperature^15^ or in the presence of amino acids,^16^ further confirming that the polymerization of cNMP follows a general base catalysis mechanism. In this process, a basic moiety deprotonates the 5′-hydroxyl group of a nucleotide, increasing its nucleophilicity and facilitating its attack on the 2′,3′-cyclic phosphate of a second nucleotide. The resulting intermediate then decays, leading to the formation of either a 3′-5′ or 2′-5′ phosphodiester bond between the two nucleotides, with the proton being accepted by the conjugate acid of the base (Figure 1b). While the analytical tools adopted here do not enable distinguishing 3′-5′ from 2′-5′ linkages, previous studies on similar systems reported an approximate 1:1 ratio between the two.^15,16^ It was also shown that backbone heterogeneity does not hamper RNA folding into functional 3D tertiary structures,^35^ making the two forms equally relevant for the appearance of ribozymes in the prebiotic world.

The reactivities reported in Figure 2a,b are markedly larger than the ones previously reported in the literature, a difference we attributed to the lower temperature and slower drying, possibly favoring supramolecular interactions. The oligomerization yield, Y, of cGMP is the highest among all single nucleotides, reaching 65% at pH 10, followed by cCMP (28% at pH 11), cAMP (17% at pH 10), and cUMP, which increased steadily with pH, up to 19% at pH 12. While G showed the highest reactivity across all pH levels, the relative reactivity of the other bases varied with pH: A > C > U at pH 10, C > A ≈ U at pH 11, and U > A ≈ C at pH 12.

Figure 2c shows the HPLC-MS analysis of the length distribution c_L_ of the products, expressed as the molar concentration of the oligomers. From preparations in the millimolar range—a prebiotically acceptable monomer concentration^30,36^ —we found at least 4-base-long homo-oligomers of all four nucleotides with concentrations ranging from 100 μM to 1 mM, and homoguanosine 10-mers at 20 μM. We found the length distribution c_L_ to generally deviate from the simple exponential decay expected for a step-growth linear polymerization,^37^ to slightly favor longer products^38^ (Figure S18). This behavior was particularly evident for G (Figures S18 and S19). For example, the polymerization of G at pH 8 generated longer products than at pH 12 despite similar Y. Similarly, C at pH 11 gave longer products than U at pH 12, and U at pH 11 gave longer products than C at pH 12. Generally, although alkaline pH is necessary to boost reactivity, a large excess of OH^–^ leads to shorter products.

To better understand the pH dependence of the reaction mechanism, we measured by HPLC-MS the amount of hydrolyzed and unreacted 2′,3′-cyclic phosphates after the reaction. Figure 2d shows the fraction of F_cP_ of the terminal phosphates for monomers and oligomers that have remained in the cyclic form and are thus potentially reactive. In other words, F_cP_ expresses the fraction of terminal 2′,3′-cyclic phosphates that have not been hydrolyzed, with its decrease by increasing pH confirming that a strong alkaline environment favors the nucleophilic attack either by the 5′OH group of another nucleotide or by a free hydroxide ion. In the former case, the reaction produces a 2′-5′ or 3′-5′ phosphodiester bond; in the latter, it yields a linear 2′- or 3′-phosphate (Figure S20). The combination of these opposing processes, cyclic phosphate opening, internucleotide phosphodiester bond formation, and phosphodiester bond hydrolysis, leads to an optimal condition for oligomerization, which appeared to be around pH 10–11 for almost all nucleotides and mixtures, U being the relevant exception, which is more tolerant to higher pH. During evaporation, the initial pH evolution depended on the nucleobases (Figure S21). In fact, guanine and uridine have self-buffering capability toward alkaline conditions because of the pK_a_ of their nitrogen atoms (N1 in G and N3 in U), allowing each RNA base to stabilize the pH in the drying process. Data in Figure 2a, however, suggest that, despite pH drifts in different ways depending on the pK_a_ of the nucleobase, the resulting oligomerization largely depends on the initial pH, indicating that additional effects come into play as the solution dries.

In Figure 2b we compare, for different pH values, the yields of AU, GC, and AUGC mixtures with the average yields of the respective single nucleotide solutions (open squares). While in general mixing appeared to reduce the yields with respect to the individual nucleobase systems, at pH 11 instead, the yields for AU and GC mixtures matched the ones expected from the average of their components, and the yield was even larger for the case of AUGC mixtures (33% at pH 11). These observations suggest that pH can tune the nucleobase composition of heterogeneous nucleobase polymerization, as discussed in detail later.

To determine the stage of evaporation at which the reaction took place, we performed HPLC analysis of products at different times, t, during dehydration. We found no relevant oligomerization in the liquid state, nor in the denser LC phase, even by stopping the evaporation to incubate the samples in these conditions for several hours (SI Figure S27). For each nucleotide, we detected the reaction products only after the dry state was achieved. Kinetics assays in the dry state indicated that for all bases the polymerization yield over time, Y­(t), followed an exponential growth with characteristic times shorter than 24 h, specifically, τ = 0.7, 2.8, 3.5, and 6.9 h for G, A, C, and U, respectively, at pH 11 (SI Figure S27).

### Effects of Supramolecular Assembly during Drying

Figure 2a,c shows that the polymerization capability of G is much larger than those of all other nucleobases. It was suggested that the enhanced activity of G is in part due to the pK_a_ = 9.3 of the N1 nitrogen,^39^ which may assist the acid–base catalysis at alkaline pH.^16^ There is, however, another element that could play a role, which is the peculiar self-assembly propensity of G nucleotides. Solutions of 5′-GMP, 3′-GMP, 2′-GMP, and 3′,5′-cyclic phosphate GMP, as well as their deoxyribose analogs 3′-dGMP, 5′-dGMP, and cyclic 3′,5-dGMP, have been found to self-assemble in G-quartets, planar structures formed by four mutually hydrogen bonded guanosines, in which N1 and N2 on the Hoogsteen edge act as hydrogen bond donors while N7 and O on the Watson–Crick edge act as acceptors.^32,34^ G-quartets, in turn, stack into linear columnar structures (G-quadruplexes, Figure 2g) that, at large enough concentrations, develop collective ordering into liquid crystal (LC) lyotropic chromonic phases.^34,40,41^ 2′,3′-cGMP was not considered in those previous studies.

Since the LC ordering gives rise to a significant optical anisotropy, we performed systematic PTOM observations on all our samples before, during, and after drying (SI Figures S22–S25). We found that, as the 2′,3′-cGMP concentration exceeds 300 mM, birefringent domains nucleated and grew to fill the whole sample with the smooth birefringent textures typical of nematic LC phases, as shown in Figure 2e (top-left panel). Upon further drying, the uniformity was lost and replaced by a faceted structure (top-right panel), indicating that birefringence, and thus long-range molecular ordering, was retained but on a smaller scale, suggesting poly-crystallinity (CRY). In our experiments, as far as PTOM observations, 2′,3′-cGMP behaved very similarly to 5′-GMP.

On the contrary, no birefringence signals of collective ordering were found in solutions of A, U, and C, which exhibit featureless dark PTOM images throughout the drying process (Figure 2e bottom panels and Figure S25), indicating a transition from an isotropic fluid to a transparent amorphous glassy state, independent of the pH. At higher pH, only the appearance of sharp-edged KOH crystals was detected. Since LC phases were previously found in binary mixtures of deoxynucleotides triphosphates,^41^ we searched for analogous structures in cyclic nucleotides mixtures, which instead we could not detect: AU mixtures showed no birefringence, while GC and AUGC mixtures exhibited only insulated birefringent domains, conceivably produced by G-quadruplex assemblies coexisting with isotropic glass regions (SI Figure S26).

On this basis, it is conceivable that the large reactivity of G is at least partly due to supramolecular ordering by which the reacting moieties are held in continuous proximity, in analogy with the proper positioning of the reacting species provided by enzymes. Indeed, LC ordering was demonstrated effective in templating nonenzymatic ligation of oligomeric DNA and RNA duplexes^42,43^ but was not reported in systems of nucleotides. At high pH,^44^ assembly of G-quartets becomes disfavored due to the deprotonation of the N1 nitrogen^39^ involved in the hydrogen bonds responsible for the assembly of G-quartets.^45^ The disruption of molecular ordering can be appreciated in the PTOM observation of droplets of the same size and same nucleotide concentration at various pH values after full dehydration, as reported in Figure 2f. This observation suggests that the peak of the reactivity of G at pH 10 may result from the combination of the appropriate phosphate opening chemistry, favored at high pH, and the geometrical arrangement provided by G-quadruplexes, favored at neutral pH. This notion is also supported by the shape of c_L_, which at pH 8 and pH 10 is clearly bimodal (Figure S19), while at larger pH, where the self-assembly is disrupted, it becomes similar to those of the other nucleotides (Figure S18).

An alternative way to disrupt G-quadruplexes is to replace counterions with Li^+^ ions (Methods),^46,47^ much smaller in size than the cavities inside the G-quadruplex structure (blue sphere in the sketch of Figure 2g) that are thus destabilized. In these conditions, the reaction yields of cGMP sharply decreased (open dots in Figure 2a) and became comparable with those of the other nucleotides. By contrast, replacement with the same ions did not significantly modify the reactivity of cCMP. These observations further confirm the notion that molecular assembly plays a role of no less importance than pH in regulating the reactivity of 2′,3′-cGMPs under the given conditions and suggest an approach to decrease reactivity differences between nucleobases, i.e., destabilize G self-assembly.

### Enhanced Polymerization Yield by Wet–Dry Cycles

Although prolonging the incubation in the dry state for t ≫ τ did not significantly impact the polymerization yield of single nucleotide systems (SI Figures S27–S28), a relevant amount of potentially reactive 2′,3′-cyclic phosphate termini were still available after 24 h, either from initial 2′,3′-cNMPs monomers or at the terminal of the produced oligomers (Figures 2d and S20), suggesting that cyclic phosphates are blocked by dehydration into positions hindering their reaction. Prompted by this observation, we periodically rehydrated and dehydrated the samples in wet–dry cycles to explore whether in a “reshuffled” dry state the remaining 2′,3′-cyclic phosphate groups could react. We performed a series of up to 10 cycles at Δt = 24 h intervals. On each cycle only pure water was added to recover the initial nucleotide and salt concentration, and a small fraction was collected for pH measurement and products analysis with HPLC and HPLC-MS (Methods).

Figure 3a,b shows the oligomerization yield as a function of the number of cycles, n, in single nucleotide solutions and in mixtures at pH ≥ 10. In all cases except for G at pH 12, Y­(n) initially grew, Y(2) > Y(1). Further cycling led to various behavior: growth of Y, saturation, or decrease depending on the system and on pH. The initially less reactive nucleotides (cCMP, cAMP, and cUMP) were found to benefit from cycling, showing a maximum yield of 33% for A at n = 7 and pH 10, and Y = 32% and Y = 25% for C and U, respectively, achieved at n = 5 and pH 11 (see also Figure S29). These nucleotides strongly benefitted from the reshuffling of their positions in the dry state, an occurrence that can take place only by fluidizing the system again by a successive hydration step. By contrast, the growth of the yield of G after the first cycle at pH ≥ 10 was limited, a behavior that we understand as a consequence of a polymerization being already very effective in the first evaporation, leaving little reactants for further improvement (Figure S29).

**Figure 3:**
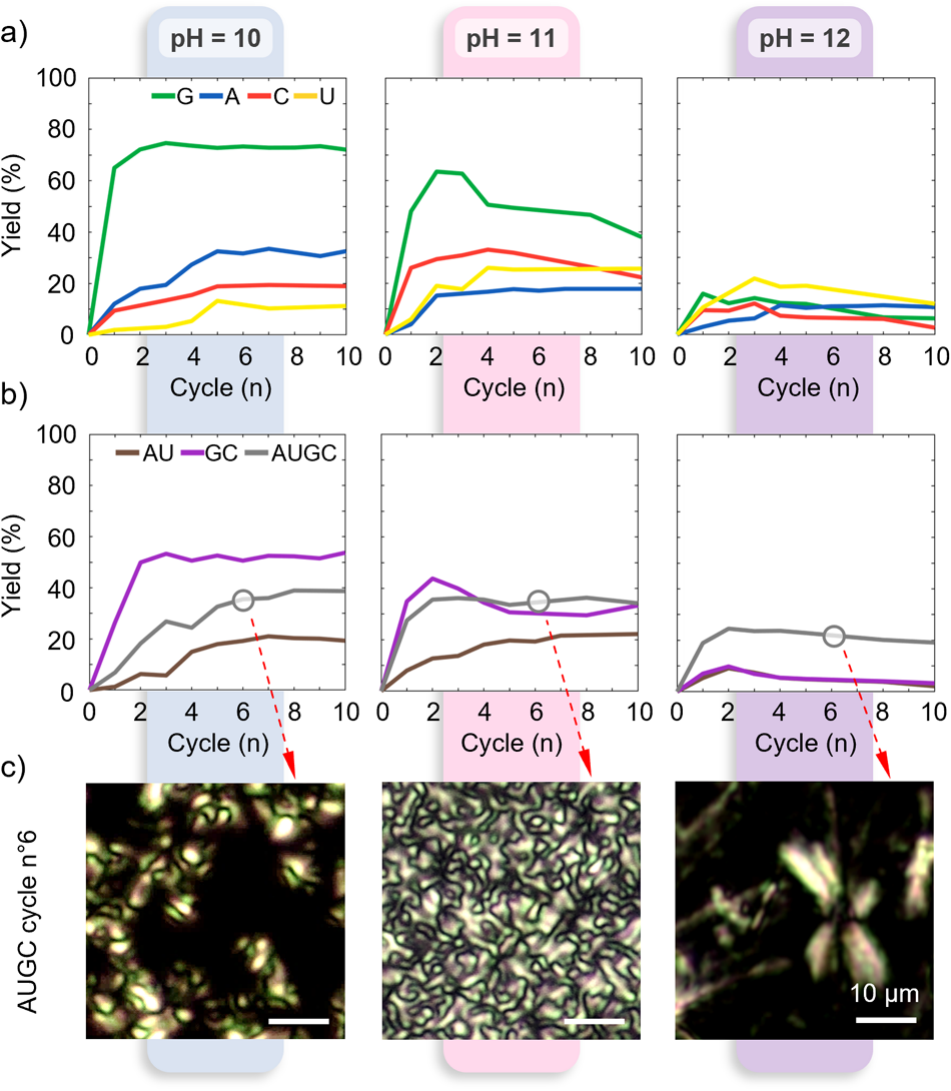
Enhanced polymerization by wet–dry cycles. a) Polymerization yield as a function of the number of cycles for 2′,3′-cNMP solutions at pH 10 (left panel), pH 11 (central panel), and pH 12 (right panel). b) Polymerization yield as a function of the number of cycles for 2′,3′-cNMP mixtures at pH 10 (left panel), pH 11 (central panel), and pH 12 (right panel). c) PTOM images through crossed polarizers of AUGC mixtures showing different birefringent domain geometries. Images were acquired after the sixth cycle in three samples with different initial conditions: pH 10 (left panel), pH 11 (central panel), and pH 12 (right panel). Yield values were obtained from HPLC analysis (Methods).

Upon rehydration cycles, we measured decreasing values of pH that converged to a constant value at n ≈ 6 for all cNMPs solutions. The average drop was ∼2 pH units, with the maximum drop being 4 pH units for solutions prepared at initial pH 10 (Figure S30). Such behavior indicated an acidification by the combined effect of 2′,3′-phosphate ring opening (Figure S29) and incorporation of CO_2_ from the environment. We found that the evolution of Y­(n) upon wet–dry cycling strongly depended on the initial pH. While cycling at initial pH 10 led to an increase or saturation of Y­(n), experiments performed at a larger pH led in some cases to a decreasing Y­(n). The decrease of Y­(n) was also evident when experiments were performed by adjusting the pH at every cycle around the optimal reactivity value that was found in cycle 1 (Figure S31). We understand this behavior as a progressive degradation of the reactive cyclic phosphates and of the produced oligomers via hydrolysis (Figure S29), the latter appearing more severe for poly-G chains.

Cycling had a strong effect on the oligomerization of binary and quaternary mixtures (Figure 3b), which reached a maximum yield of 53% for GC at pH 10, 22% for AU at pH 10, and 36% for AUGC at pH 11. With increasing n, the yield of the mixtures was found to generally approach the average of the yield of the relative single nucleotide systems (SI Figure S32), with the only exception AU at pH 12. Moreover, the yield of the AUGC mixture at pH ≥ 11 overcomes those of CG and AU mixtures and of their averages (Figures 3b and S32), suggesting that under these conditions heterogeneous polymerization is larger than the homogeneous one.

Consequently, also the products distributions reflected the reactivity enhancement caused by periodic rehydration (Figure 4). HPLC-MS analysis enabled us to detect RNA oligomers up to 10 μM and 4 μM 10-mers in GC and AUGC mixtures, respectively, or up to 0.15 mM 5-mers in AU mixtures after 10 cycles at an initial pH 10. Concentrations, c, are expressed in terms of the initial nucleotide concentration (50 mM) and correspond to a mass fraction of these oligomers of 0.2%, 0.1%, and 1.5%, respectively. At pH 11, despite the expected competition due to hydrolysis, increasing with n, production of 2-mers and 3-mers at mM concentrations was observed in all mixtures, with maximum detected lengths of 0.2 mM 4-mers in AU mixtures at n = 10, 50 μM 7-mers in GC mixtures, and 30 μM 8-mers in AUGC mixtures (i.e., 1.6%, 0.7%, and 0.5% mass fraction).

**Figure 4:**
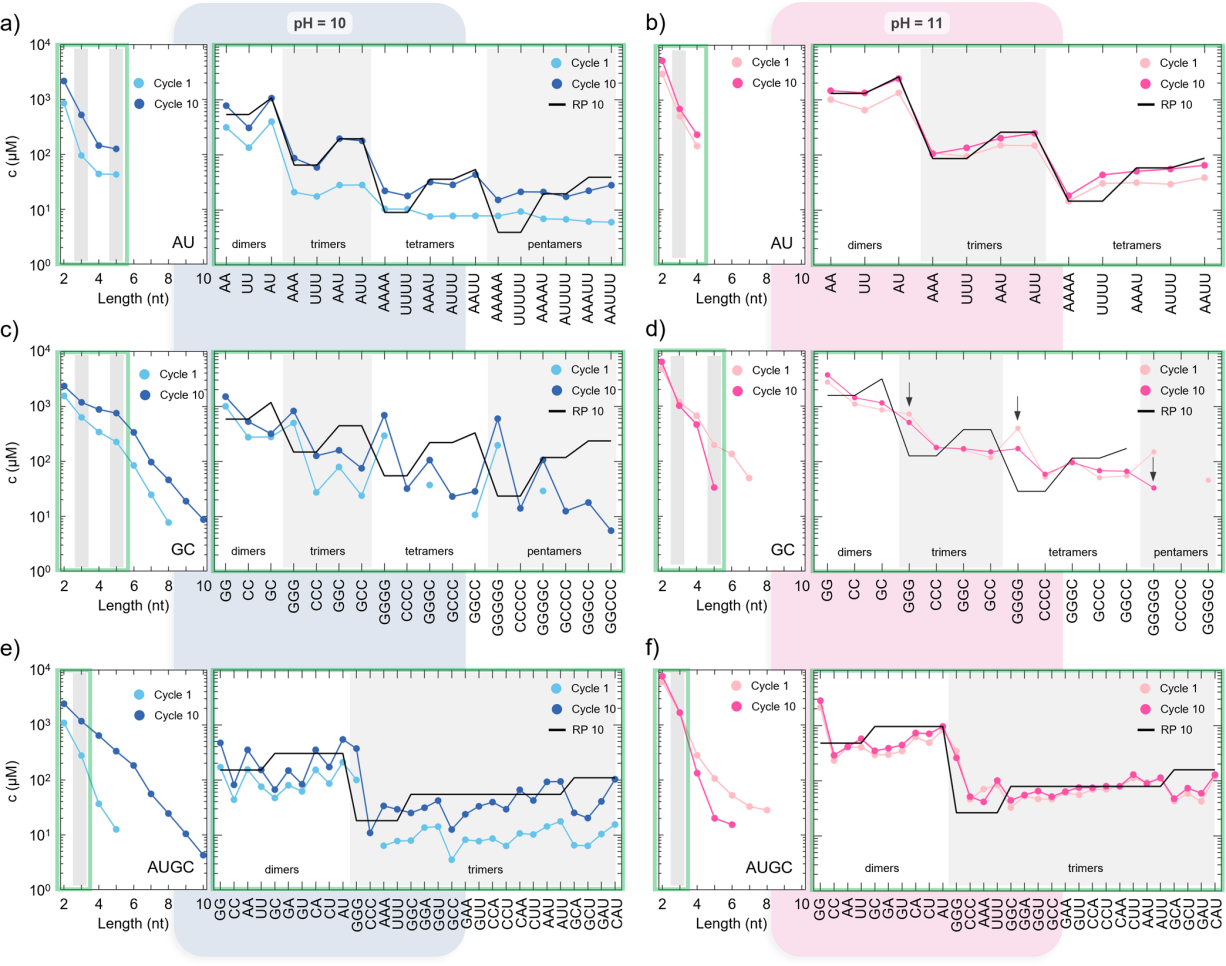
HPLC-MS analysis of nucleobase composition for products formed in AU, GC, and AUGC mixtures. Panels a–f show the length and sequence distributions of oligomers formed in different mixtures after the first and 10th wet–dry cycles. Panels a–e display results at pH 10 (marked by a blue ribbon). Panels b, d, and f display results at pH 11 (pink ribbon). In each panel, the left plot displays the product length distribution, while the right plot shows detailed sequence compositions in the length interval marked by the colored frames. Within frames, alternating vertical white and gray shading helps to distinguish oligomers according to their length. a,b) Length and sequence distribution analysis for AU mixtures at pH 10 (a) and pH 11 (b). Black lines represent the expected distribution after the 10th cycle in the case of random polymerization (RP), normalized to the experimental concentration for each oligomer length. c,d) Same as above for GC mixtures. Arrows mark the oligo-G sequences whose population is reduced by cycling because of the cleavage of the phosphodiester bonds. e,f) Same as above for AUGC mixtures.

### Enhanced Nucleobase Heterogeneity by Wet–Dry Cycles

A basic requirement for the RNA world is the production of chains with the significant degree of heterogeneity in their nucleobase sequence that is required to promote sequence elongation^43,48^ and replication^49,50^ through base pairing and nonenzymatic ligation. Thus, for the spontaneous polymerization of 2′,3′-cNMPs by wet–dry cycling to be a source of building blocks of longer chains, it needs to offer a large palette of nucleobase alternation along the chain. Our results show that, if not mitigated via alkaline conditions, the larger reactivity of G might lead to G-dominated oligomers,^15,16^ in which the strong association of the G-rich sections would hinder the access to secondary structures based on Watson–Crick pairing. A balanced incorporation of the different nucleobases in the produced RNA oligomers would likely require at least two conditions to be met: equalizing the reactivity of all four nucleotides and granting homogeneous spatial distribution of the reactants to ensure equal probability of contacts among different species.

Our data suggest that these conditions can be achieved at pH ≥ 11 and increasing n, where the polymerization yields of individual A, U, G, and C become similar (Figure 3a and SI Figure S33). At the same time, PTOM observations of the dry state of AUGC mixtures revealed that the self-assembly capability of the system is also modified during wet–dry cycles, driving the formation of different morphologies (e.g., at n = 6, Figure 3c). Indeed, at pH 10 we observed the coexistence of crystals and amorphous glass, which we understand as a phase separation between a G-rich crystal phase and other nucleotide species. This interpretation agrees with the observation of a similar coexistence in GC mixtures, in the same conditions, while only an isotropic amorphous glass phase is observed in AU mixtures or in the absence of G (SI Figures S25, S26, S34). Conversely, at pH 11 much more homogeneous crystalline phases are observed throughout the whole sample, suggesting a form of ordering in which all polymerization products participate (SI Figure S34).

To probe whether the combination of equalized reactivity of different nucleotides and a homogeneous dry state can influence the degree of chain heterogeneity, we analyzed by HPLC-MS the nucleobase composition of the RNA oligomers produced after 1 and 10 wet–dry cycles of cNMPs mixtures, with initial pH 6–12 (Figure 4, SI Figures S35–S40 and SI Methods).

AU mixtures were characterized by similar polymerization yields of individual A and U systems for pH ≥ 11 (Figures 3a and S33) and by homogeneous isotropic amorphous glass dried states at each pH (Figure S34). Figure 4a,b shows the concentration, c, of each combination of A and U in chains of length 2–5 at pH 10 and pH 11. In this analysis, the ordering of the sequences cannot be distinguished by the mass spectrometry, e.g. “AAU” ≡ {AAU, AUA, UAA}. The results are compared with the expected product distribution for random polymerization (RP) based on the combinatorics of reshuffled RNA sequences with the same length (black line) with the total concentration equal to the sum of the measured concentrations of the produced oligomers of the considered length (see SI Methods and Figure S38). At pH 11 we found a good agreement between the measured c and the distribution expected for random polymerization, with the only exception of UUUU excess (see also Figure S41). This result indicates that the self- and mutual reactivities of A and U are similar. Cycling at pH 11 increases the yield but does not significantly change the product distribution. At pH 10, instead, cycle 1 and cycle 10 are markedly different, initially dominated by homonucleotide products, slightly favoring A-rich products over U-rich ones, and later compensated by a larger growth of heteronucleotide polymerization events. This suggests that the molecular arrangement in the dry state could be affected by the presence of short oligomers in the preceding fluid solution.

GC mixtures were instead characterized by a strong difference of polymerization yields for individual G and C systems (Figures 3a and S33), which decreases only for pH ≥ 11 or large n, and by ISO-CRY phase separation, at each pH and n (Figure S34). The distribution of oligomers produced in wet–dry cycles of CG mixtures were indeed found to be dominated by poly-G sequences, due to the larger reactivity of G, as apparent in Figure 4c,d (see also Figure S39). As expected from the yield measurements (Figures 3a, S33), this effect was much stronger at pH 10. At this pH, the distribution of products grew in amplitude from cycle 1 to cycle 10, but its uneven character remained. At pH 11 we observed a flatter distribution which became closer to the distribution expected for random polymerization as the number of cycles increased (Figure S41). Inspection of the products revealed that this is the consequence of a minor increase of heterogeneous polymerization and of a significant decrease of the number of poly-G chains longer than 3 nucleotides (arrows). Such a decrease is in line with the reduced yield in G solutions upon cycling at pH 11 (Figure 3a) and with the cleavage of longer poly-G (arrows in Figure 4d) when cycles were performed in a controlled highly alkaline environment (Figure S30). The dominance of poly-G chains suggested that a residual phase separation due to the self-assembly propensity of G was still present at each pH and n, making the physical proximity of C and G less likely, in agreement with PTOM observations (Figure S34).

The product distribution in the AUGC mixture (Figure 4e, f) appeared to be a combination of those of the two binary mixtures. At pH 10 the inclusion of all four nucleotides was found to be uneven throughout all cycles, in agreement with the larger reactivity of G (Figures 3a and S33), and mixed products including G (e.g., GCC, GAA, and GUU) tended to have lower concentrations than mixed products without G (e.g., CAA, CUU, and AUU), in line with the presence of phase separation (Figure 3c). At pH 11, we observed a rather flat base sequence distribution in short oligomers (Figure S40), which correlates with the expected decrease in the reactivity difference between G and the other nucleobases (Figure S33), the enhanced cleavage of long poly-G (SI Figure S29), and the suppression of phase separation (Figure 3c) upon cycling. This reshuffling mechanism, driven by a combination of oligomer growth, via residual cyclic-phosphate transesterification, and oligomer breakdown, through hydrolysis reactions (SI Figure S37), led to a stable production of well-mixed short oligomers after 10 wet–dry cycles, indicating a possible self-regulating pathway for the prebiotic production of randomized RNA oligomers with all four nucleobases.

At pH 12, the sequence distributions of products for AU, GC, and AUGC exhibited an even closer alignment with the expected distribution for random polymerization (Figures S41–S42). This observation is consistent with the more uniform reactivity among the nucleotide species under these conditions (Figure S33). However, the oligomer lengths were significantly constrained due to increased hydrolysis at high pH (Figures S35–S37).

## Conclusions

We have demonstrated that the long-standing problem of abiotic polymerization of RNA under prebiotically plausible conditions can be approached by unassisted polymerization of 2′,3′-cNMPs prepared at mM concentration in salt-depleted aqueous solutions that undergo periodic dehydration and hydration at room temperature. Polymerization occurred under mildly alkaline pH conditions without the addition of catalysts or external activators that would reduce its likelihood. Alkaline conditions are found today in the surface waters of volcanic islands^51^ and alkaline lakes^52^ and would likely have been similar on prebiotic Earth. Such an environment would also implement wet–dry cycles through day–night cycles as well as differences in weather conditions with both pH buffered and unbuffered cycles. For example, an initial alkaline spring could provide the starting pH, while wet–dry cycles might occur either in the same setting, where rock minerals actively buffer the alkaline pH, or in a different environment with distinct minerals. In the latter case, rehydration could be driven by rainwater, which was likely acidic due to the high CO_2_ content of the early Earth’s atmosphere,^53^ allowing for transient pH shifts.

Under these conditions, we found superior polymerization yields and a wide variety of RNA sequences containing all four nucleotides. We interpreted the larger reactivity of G as a consequence of its natural tendency to self-assemble into ordered G-quadruplex columns. Once these structures were destabilized by pH or G-quartet adverse ions such as Li^+^, the reactivity of G significantly decreased, enabling equalization of the reactivity among nucleobases. Different combinations of pH and cycling were sufficient to generate a nascent RNA population in equimolar solutions of the four nucleotides, with moderate alkaline pH (8–10) favoring longer (10-mers), G-dominated oligomers and more alkaline conditions (pH 11–12) favoring shorter (8-mers), more compositionally diverse sequences (Figure S42).

We found that pH 11 provided an optimal trade-off, with 36% yield in AUGC mixtures and detection of 8-mer RNA oligomers at a concentration of 30 μM (Figure 4f). Under these conditions, compositional diversity in terms of nucleobase sequence in the polymerized RNA chains is favored. The reported polymerization of RNA under wet–dry cycles ensures the availability of relevant concentrations of oligomers capable of stable pairing over a wide temperature range,^54^ providing a solid basis for initiating early RNA evolution.^55^ The complementary alternation of dilute-phase reshuffling and concentrate-phase self-assembly,^56^ together with the regeneration of 2′,3′-cyclic phosphate termini,^9^ could lead to the transition to different mechanisms of nonenzymatic oligomer polymerization^42,43^ and templated ligation,^50^ promoting further elongation and information copying of the seeding random-sequence RNA population obtained here.

## Materials and Methods

### Materials

2′,3′-Cyclic phosphate (2′3′-cNMPs), 2′-phosphate (2′-NMPs), and 3′-phosphate (3′-NMPs) nucleotides were purchased from BioLog (Germany) in their sodium salt form. Oligonucleotides were purchased from Biomers (Germany), and chemicals were from Merck (Germany).

### Stock Solutions and Samples Preparation

Diluted solutions (∼10 mM) of single 2′3′-cNMP species (cAMP, cUMP, cGMP, cCMP) and equimolar binary and quaternary mixtures (AU, GC, AUGC) were prepared in Milli-Q water (Millipore), portioned in 200 μL plastic tubes (Eppendorf), corresponding to 1 μmol of nucleotides in each tube, and then lyophilized overnight. The resulting powders were stored at room temperature. Samples were prepared resuspending each portion in 20 μL of Milli-Q water to achieve the initial concentration c_0_ = 50 mM, and the pH was adjusted using potassium hydroxide (KOH). Solutions were evaporated into a flat glass-bottom multiwell plate (Corning) in open air at room temperature over 24 h. On average, all samples achieved the dry state within 8–10 h. Evaporations of 2 μL solutions were also performed on a glass microscopy slide. In this case, due to the lower volume and the open geometry, the evaporation was faster (10 to 20 min) but the oligomerization yields were found to be similar. Measurements of the concentration during evaporation were performed by weighing 20 μL of 2′3′-cNMPs solutions in a plastic tube (Eppendorf), over time, with a scale with 0.01 mg sensitivity (ABT 100-5M from Kern).

### Ion Exchange

Contrarily to K^+^, the affinity of Li^+^ ions for G-quadruplexes has been reported to be lower than that of Na^+^.^32^ Therefore, complete replacement of Na^+^ counterions with Li^+^ ions was needed to probe the effect of Li^+^ ions. This was done through the following ion-exchange protocol. 600 mg of resin (Dowex 50WX8 hydrogen form) was placed in a 2 mL plastic tube (Eppendorf) together with 1 mL of 2 M solution of LiOH and stirred with a magnetic stirrer. After 5 h, if pH was <7, the supernatant was replaced with fresh 2 M LiOH and the solution was further stirred for 5 h. The Li^+^-enriched resin was filtered and loaded in a 1 mL syringe. 300 μL of a 30 mM solution of cyclic nucleotides was loaded on top of the resin, eluted, and collected in a 2 mL plastic tube. The resin was further washed with 1 mL of Milli-Q water and collected in the same tube. The obtained solution was promptly flash-frozen and lyophilized. The replacement of Na^+^ with K^+^, following the same procedure, did not produce different behavior compared to the direct addition of KOH to sodium salt cNMPs.

### Microscopy Observation

Brightfield and polarized transmitted optical microscopy observations were performed with an inverted optical microscope (TE200 from Nikon, Japan), equipped with 4x, 10x, 20x, and 50x magnification objectives. Images were acquired with a Nikon DS-Fi3 CMOS color camera, controlled by NIS-Elements BR software (version 5.11.03).

### Wet–Dry Cycles and Sampling Protocol

Samples were rehydrated every 24 h by adding 20 μL of deionized water and without any pH adjustment and allowed to equilibrate for 10 min before withdrawing any material for analysis. Optical microscopy observations confirmed the dissolution of any solid residues and that the rehydrated solutions turned back into the homogeneous diluted phase. At each cycle, 0.3 μL samples of the rehydrated solutions were collected for immediate pH measurement with pH-indicator strips (Merck), and 0.6 μL samples were collected and lyophilized for HPLC and/or LC-MS analysis. The analyzed aliquots were representative of the entire system, extracted from a diluted homogeneous solution. All experiments were performed in 2 independent replicates.

### HPLC Analysis

High-performance liquid chromatography (HPLC) analyses were performed with a WaveTransgenomic (Hitachi) instrument equipped with an Xbridge BEH C18 OST column (4.6 × 50 mm, particle size = 2.5 μm) and a deuterium lamp (Hitachi, 892-2550). The instrument was calibrated with a ladder of oligomers for each nucleotide species (3′-phosphate ending 1-mer, 2-mer, 4-mer, and 10-mer). 100% TEAA (bottle A) and 75% TEAA–25% acetonitrile (bottle B) were used as solvents. Each extracted reaction sample was diluted to a final concentration of 1 mM with Milli-Q water. Then, 10 nmol samples of nucleotides (10 μL) were injected into the HPLC column previously equilibrated at 65 °C. Once the temperature was stable, the flow rate was set at 1 mL/min to let the column equilibrate for 3 min at 100% A. Then the following step gradient was performed: 0% to 60% B in 15 min, 50% to 46% B in 2 min, 46% to 0% B in 1 min. UV absorbance was measured at λ = 260 nm. See Supporting Methods for chromatogram analysis.

### Yield and Product Concentration Calculations

Oligomerization yield (Y) is defined as the percentage of the initial nucleotides that are converted into oligomers, namely the ratio between oligomerized mass over total mass of starting material. Reaction yield was computed, through integration of the identified products peaks of HPLC-UV or LC-MS chromatograms, as

$$Y=\frac{\mathrm{oligomerized}\;\mathrm{mass}}{\mathrm{total}\;\mathrm{mass}}=\frac{\underset{L\geq 2}{\sum }A_{L}}{\underset{L\geq 1}{\sum }A_{L}}$$

where *A*_L_ is the area of the peaks attributed to the oligomers of length *L*. The product distributions were obtained by calculating the concentration (c_L_) of each oligomer of length L. To compute c_L_ we multiplied the yield of oligomers of length *L* ( $Y_{L}=\frac{A_{L}}{\underset{L\geq 1}{\sum }A_{L}}$ ) by the total concentration of starting material (50 mM). Further information about yield and concentration calculations is reported in the Supporting Methods.

### LC-MS Acquisition Method

The samples were received in a lyophilized state and then rehydrated in RNAse-free water (Thermo Fisher Scientific). 10 nmol (for individual nucleotide samples) to 40 nmol (for cAUGC samples) portions were injected in the HPLC for analysis, without further treatment for the majority of the samples.

Measurements were performed on an HPLC (Agilent 1260 Infinity II) coupled to an electrospray ionization time-of-flight (ESI-TOF) mass spectrometer (Agilent 6230B with dual AJS ESI). The column used was an Agilent Advance Oligonucleotide C18 column (4.6 × 150 mm 2.7-μm) heated at 60 °C, and the oligomers were separated by using length ion-pairing reverse-phase HPLC. The eluent consisted of mixtures of water (Bottle A) and methanol (Bottle B: 50% water, 50% methanol), each containing 8 mM triethylamine (TEA) and 200 mM hexafluoro­isopropanol (HFIP), with a gradient elution at a flow of 1 mL/min. The method started with 1% of B for 5 min, followed by a gradient, increasing from 1% to 30% B over 22.5 min and then to 40% for 15 min. Then, the column was flushed with 100% B for 5 min before being returned to 1% for 6 min to re-equilibrate the column.

Detection of eluted mononucleotides and oligonucleotides was achieved using ESI-TOF in negative mode (employing specific source parameters: Gas temperature: 325 °C; drying gas flow: 13 L/min; sheath gas temperature: 400 °C; sheath gas flow: 12 L/min; VCap: 3500 V; nozzle voltage: 2000 V); and Diode Array Detector (DAD) WR (wavelength used: 260 nm).

## Supplementary Material



## Notes

Additional information about products identification and quantification, experimental details, reaction and dry phase characterization, methods and sequence composition analysis (PDF)
