# Supplementary material for: High-Yield Prebiotic Polymerization of 2′,3′-Cyclic Nucleotides under Wet–Dry Cycling
Source: ACS Cent Sci. 2025 Jun 28;11(9):1546–57. doi: 10.1021/acscentsci.5c00488 (PMC12464751; doi:10.1021/acscentsci.5c00488)
Supplement: Supplementary file 1 [file oc5c00488_si_001.pdf]

High-yield prebiotic polymerization of 2′,3′-cyclic nucleotides under wet-dry cycling

Federico Caimi[1,°], Juliette Langlais[2,°], Francesco Fontana[3,°], Sreekar Wunnava[2], Tommaso Bellini[1,*], Dieter Braun[2,*] & Tommaso P. Fraccia[3,*]

° Joint first authors; * Joint corresponding authors;

tommaso.bellini@unimi.it, dieter.braun@lmu.de, tommaso.fraccia@unimi.it

[1] Dep. of Medical Biotechnologies and Translational Medicine, University of Milan, via Fratelli Cervi 93, I-20090 Segrate, Milano, Italy

[2] Systems Biophysics, Ludwig-Maximilians-University Munich, Amalienstr. 54, 80799 Munich, Germany

[3] Dep. of Pharmacological and Biomolecular Sciences, University of Milan, via Balzaretti 9, I-20134 Milano, Italy

Supporting Information

Table of Contents

Supporting methods......2

Supporting figures......19

Supporting references......45

Supporting methods

HPLC calibration and yield measurement

The HPLC setup was calibrated by individually injecting solutions of oligonucleotides with 3'-phosphate ending (dimers, trimers and tetramers from Biomers). A baseline of MilliQ water was used to correct each chromatogram. Absorbance peaks were numerically integrated with a trapezoidal method and attributed to the different reaction products by comparison with the calibration oligonucleotides traces. Depending on experimental conditions (environment temperature and buffer preparation), we observe differences in retention times within 1 minute. The reaction yield was calculated as:

$$Y = \frac{polymerized\ mass}{total\ mass} = \frac{\sum_{n\geq 2} A_n}{\sum_{n\geq 1} A_n}$$

where A_n is the area of the absorbance peaks attributed to the oligomers of length *n*. When computing the yield, the distinction between linear and 2',3'-cyclic phosphate termini is not considered.

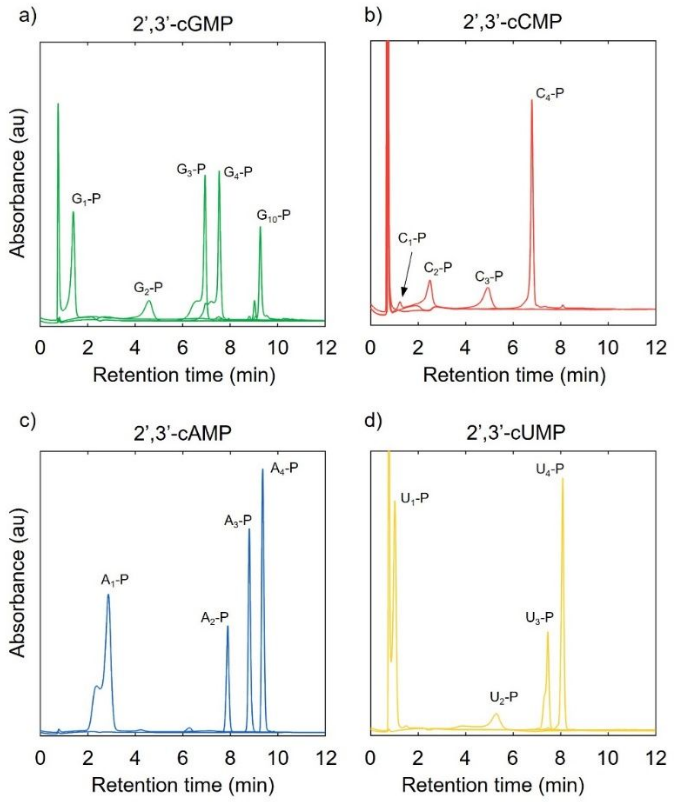

Figure S1. HPLC traces of oligonucleotides with definite sequence and length for calibration. a) poly-G standards, b) poly-C standards, c) poly-A standards, d) poly-U standards.

Product identification and quantification by LC-MS

The complete procedure is described in detail in the following sections: *"LC-MS calibration and analysis"* and *"MS data analysis by custom-written LabVIEW program"*. The calculations for concentrations and yields are presented in the section *"MS determination of reaction yield and product concentrations"*.

To summarize, product identification is based on mass spectrometry detection, supported by LC separation according to product length (see Figures **S2,3**). The two main identification steps are:

1. In the mass chromatogram of the product, we check for a peak at the expected retention time, based on comparison with a standard of the same length (Figure **S3**).
2. In the spectrum of the peak, we verify the full isotopic distribution expected for the molecule (as shown in Figure 1d of the main text). This step ensures that the detected mass in step 1 is not due to an isotope of a different molecule (see also Figure **S13**).

Quantification is then performed using standard monomers and oligomers of corresponding lengths.

LC-MS calibration and analysis

Samples for LC-MS analysis were lyophilized, stored and then rehydrated prior to injection in the instrument. This allowed us to get a more detailed view of the products: their composition (G/C/A/U) and the hydrolysis of the 2′,3′-cyclic ending into linear 2′ and 3′-phosphate.

Standards and separation on the column

Standard pre-synthesized oligomers of G (2 to 10mers), C (2 to 10mers), A (2 to 4mers) and U (2 to 4mers) with a 3′-phosphate ending (and -OH at the 5′ end) were purchased from biomers.net as lyophilized samples. G, C, A, U monomers with 2′,3′-cyclic phosphate, 2′-phosphate and 3′-phosphate were purchased from BioLog – Life Science Institute as lyophilized samples. The standards were rehydrated in RNAse-free water and stored at -80°C.

The standards monomers and oligomers are well separated on the column (see Figure SM2). They are used for the quantification in the following sections (Monomers quantification and Oligomers quantification). It needs to be noted in the Figure **S2** that the species G/C/A/U-P correspond to a 1:1 mix of the 2′-phosphate and 3′-phosphate mononucleotide. Those two molecules elute at the same time with the LC acquisition method used (see LC-MS acquisition method section in the main article) and they have the same mass. Thus, they were never differentiated in the analyses and were quantified together.

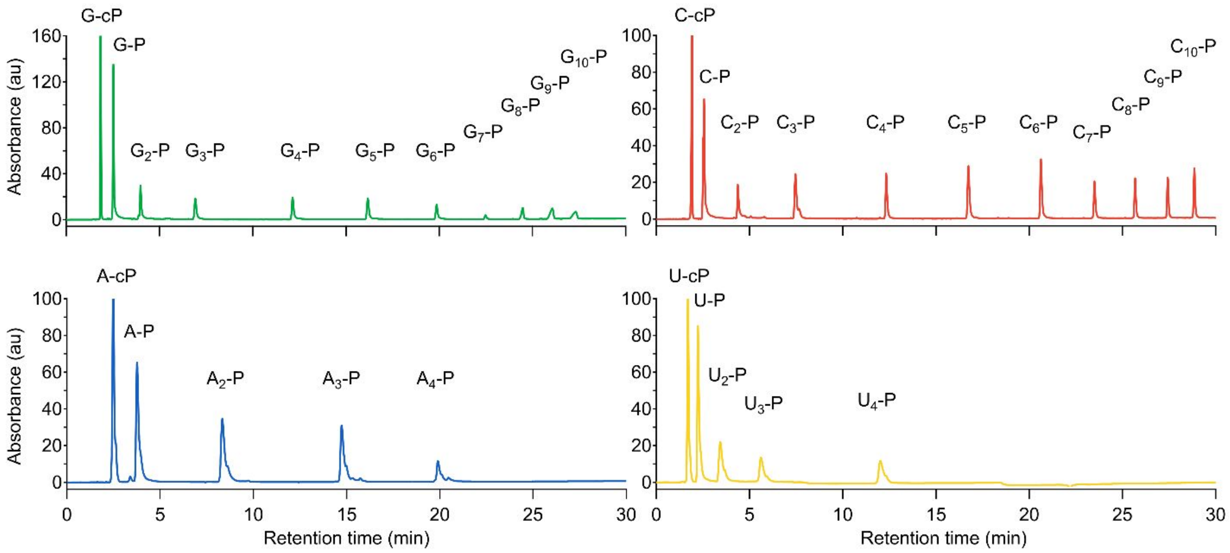

Figure S2. LC-MS calibration traces for G, C, A and U standards. The traces correspond to the absorbance at 260 nm. Quantification coefficients were obtained using G, C, A, U monomers (N-cP: 2'3'-cyclic and N-P: 2':3'-linear 1:1 mix) and oligomers standards with a 3'-phosphate ending: G (2 to 10), C (2 to 10), A (2 to 4) and U (2 to 4).

It needs to be noted that oligonucleotides with a 3'/2' phosphate ending (-P) with a length *n* elute at a similar retention time to cyclic phosphate ending (-cP) oligomers of length *n+1* (see Figure SM3).

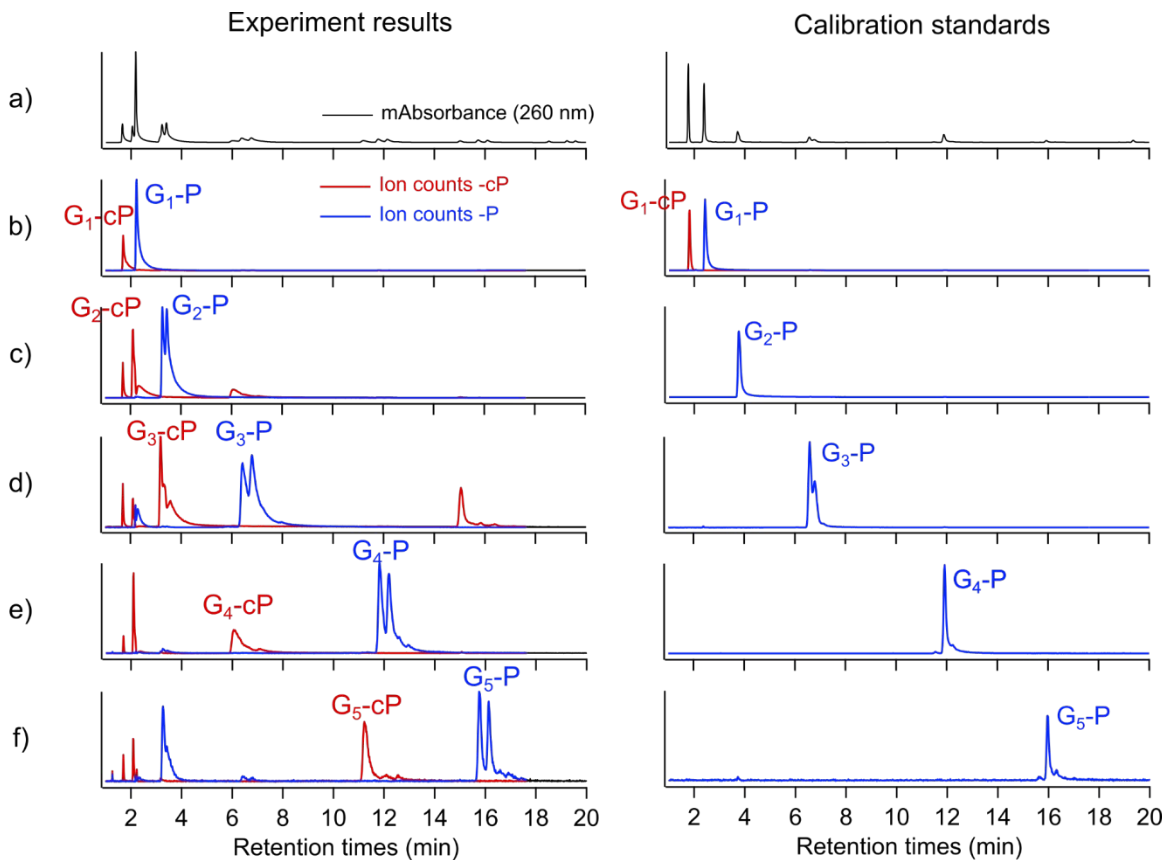

Figure S3. Comparison of G mono- and oligomers retention times between experimental results and the calibration standards. The ion counts chromatograms corresponding to the 2'/3'-P ending oligomers are in blue and the ones corresponding to the 2',3'-cP ending oligomers are in red. The experimental data (left) were extracted from a G polymerization sample at pH 10 after 10 wet-dry cycles. The calibration standards shown right were run the same day to quantify the experimental data. a) UV absorbance chromatogram at 260 nm. Extracted Ion Counts (EIC) chromatograms for the m/z values b) 344.04 (G-cP) and 362.05 (G-P); c) 689.09 (G_2-cP) and 707.10 (G_2-P); d) 1034.14 (G_3-cP) and 1052.15 (G_3-P); e) 1379.18 (G_4-cP) and 1397.19 (G_4-P); f) 1724.23 (G_5-cP) and 1742.24 (G_5-P).

Monomers quantification

Problem with calibration using ion counts

The absorbance values were obtained using the Agilent software MassHunter Qualitative Analysis Navigator B.08.00. For the analysis on the LC-MS, around 10 nmol of each sample are injected (around 20 nmol for GC or AU samples and around 40 nmol for AUGC samples). Therefore, for the quantification of the remaining monomers, the standard calibration curves were plotted for the range [10 ; 0.1] nmol. But, as can be seen in Figure **S4**, the ion counts of the monomers are not linearly dependent on the quantities for that range. Thus, unlike for the oligomers, the ion counts were not used for the quantification of the monomers with a cyclic ending and a hydrolyzed 2'/3'-phosphate ending.

Standards solution and calibration by integration of the UV absorbance peak

The monomers (cyclic and linear-ending) were quantified by numerically integrating the corresponding UV absorbance peaks at 260 nm using the Agilent software MassHunter Qualitative

Analysis Navigator B.08.00. For each nucleobase (N = G, C, A or U), a standards' solution of 100 µM N-cP and 100 µM N-P (50 µM 2'-NMP + 50 µM 3'-NMP) is prepared and 1, 2, 3, 6, 10, 20, 30 and 100 µL of the solution were injected. The calibration curves are plotted for the range [0.1; 0.2; 0.3; 0.6; 1; 2; 3; 10] nmol.

Quantification method for the remaining monomers in the polymerized sample

As can be seen in Figure **S2**, the cyclic-phosphate monomers elute first and are well separated from the other species. The linear phosphate monomers (N-P) elute at the same retention time as the cyclic-ending dimers (N_2-cP) (see Figure SM3). Thus, the quantification of the resulting UV peak gives a total quantity of nucleotides [N] = [N-P] + 2*[N_2-cP]. The distinct quantity of the dimers N_2-cP is obtained by quantification using their ion counts (see Polymers quantification section) and is removed from the total monomer concentration [N]. The remaining nucleotide quantity [N] - 2*[N_2-cP] corresponds to the linear phosphate ending monomers [N-P].

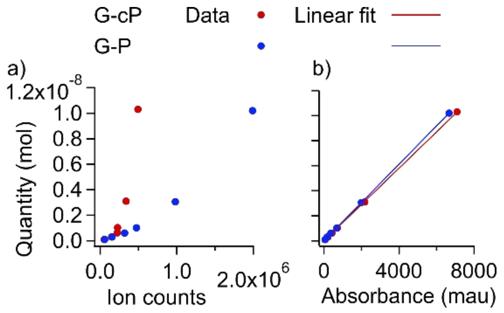

Figure S4. Comparison between the calibration curves for G monomers using ion counts and the absorbance at 260 nm. a) The plotting of the ion counts against the injected quantities of the monomers 2',3'-cGMP (G-cP) and 2'/3'-GMP (G-P) shows a nonlinear behavior for the range [10 ; 0.1] nmol (idem for the A/U/C standards monomers). The ion counts were obtained using a custom-written LabVIEW program which workflow is described in the following section. b) Calibration curve for the monomers 2',3'-cGMP (G-cP) and 2'/3'-GMP (G-P) using the absorbance at 260 nm. The coefficients used for the quantification of the monomers in the polymerization experiments are the slopes of the linear fit of the calibration data.

The detection limit for monomers on the HPLC-MS was found to be around a few 10 pmols.

Polymers quantification

Standards solution

Before each new series sample analysis, the standards solutions were also injected to plot the calibration curves (the ionization of the molecules and therefore the value of the coefficients is dependent of the state of the MS). The standards' solutions were prepared for each nucleobase (2-10mer for G and C and 2-4mer for A and U) with the concentrations in the table SM1 and different volumes were injected: 1, 2, 3, 6, 10, 20, 30 and 100 µL.

Table S1. Standard oligomers concentration for the calibration solutions:

n-mer	2	3	4	5	6	7	8	9	10
C (µM)	40	20	10	10	10	5	5	5	5

Calibration by ion counts

As seen in Figure **S5**, the ion counts are linearly dependent on the quantities for the oligomers, within the range of injected quantities. Therefore, ion counts were used for the quantification of the oligomer products for the LC-MS analysis method. The ion counts were obtained using a custom-written LabVIEW program which workflow is described in the section (MS data analysis by custom-written LabVIEW program).

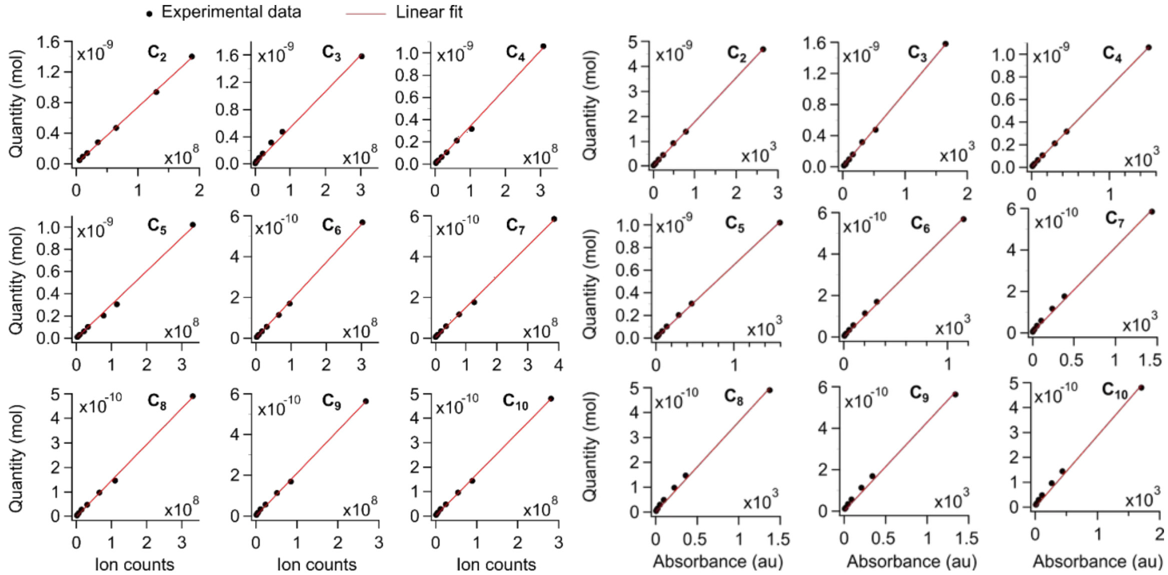

Figure S5. Example of calibration curves for the standards C oligomers 2 to 10mers, with the injected quantities plotted against the ion counts (left set) and absorbance at 260nm (right set). Unlike for the monomers, the calibration curves of the oligomers are linear for the quantities ranges that were detected in the polymerization experiments both in ion counts at the MS and the UV absorbance. As both UV and MS fall in the linear range, ion counts were used for the quantification of oligomer products (same apply for the A, U and G standards). The ion counts are a result of fitting the isotopic distribution and can distinguish different oligonucleotides. Unlike the standards, the UV peaks for the samples are not peaks of a single molecule and can only be used to estimate the total yield and not the concentration of various oligomeric species. For each length of oligomer, the calibration coefficient corresponds to the slope of the linear fit (in red). A new calibration was done for each new series of analysis, as the values of the coefficients changed with the instrument and the state of the instrument.

Quantification method for the synthesized oligomers in the polymerized sample

If detected, the oligomers of length between 2 and 10 nucleotides were quantified in the polymerization samples. Products of length > 10 were not quantified. The standards used were all with a linear 3'-phosphate ending. The coefficients correspond the slope of the linear fit of the calibration data (see Figure **S5**). Cyclic ending oligomers were quantified using the coefficient of the 3'-phosphate ending standard of the same length. Mixed products obtained in AU mixtures were quantified using A standards. Mixed products obtained in GC or AUGC mixtures were quantified using C standards. The slight variations due to the nucleobase composition are negligible for the oligomers as the phosphate

is the molecular group that contribute the most to the ionization of the molecule in the MS with the parameters of the acquisition method (Materials and Methods in the main article). In the case of mixed oligomers, it must be noted that the LC-MS method allows only for the detection of the product composition and does not differentiate between two permutations of the same combination (e.g. GC and CG).

The detection limit for monomers on the HPLC-MS was found to be around a few pmols.

MS data analysis by custom-written LabVIEW program

Introduction

The standard method of using mono-isotopic m/z and integrating the extracted MS chromatogram could not be applied for the analysis of mixed oligomerization of A, U, G and C due to considerable overlaps in the isotope distributions of different sequences of lengths (and phosphate ending) *n*-P and *n+1*-cP (see Figure SM3). This would result in wrong estimation of concentrations (see Figure SM6).

For example, one typical overlap is the one between C and U monomers (and oligomer products): as shown in Figure SM6 a) and b) for the molecules GGC-P and GGU-P, their isotopic distributions are separated by only 1 unit. Thus, the first isotope of U corresponds to the second isotope of C. A more detailed list of possible overlaps between AUGC sequences can be found in an already published work [1].

To circumvent this problem, the different oligonucleotides were quantified based on the fit of the isotopic distributions of all the possible molecules at any given retention time (see MS data analysis pipeline section).

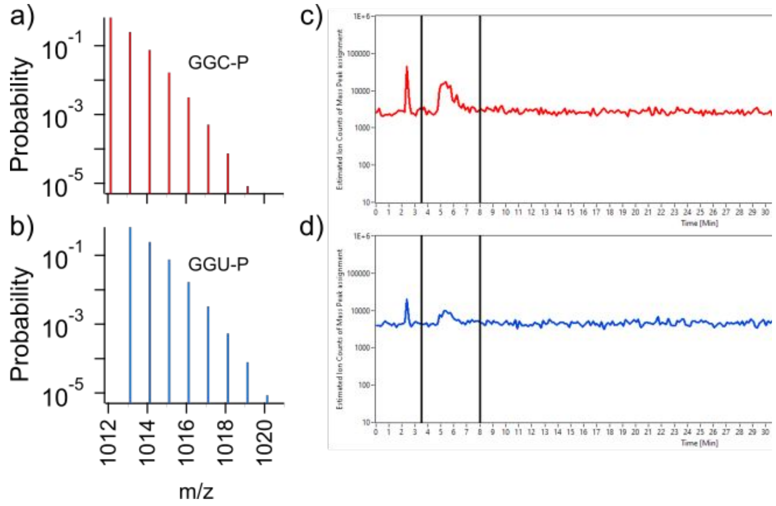

Figure S6. Example of overlap: GGC-P and GGU-P. a) Isotopic distribution (1st charged state = ionization by removal of 1H from the neutral, fully protonated molecule) of GGC-P (GGC oligomer with a linear phosphate ending). b) Isotopic distribution of GGU-P. c) Extracted Ion Count (EIC) chromatogram for the mass 1012.14 – corresponding to the first isotope of GGC-P – over time. d) EIC chromatogram for the mass 1013.12 – corresponding to the first isotope of GGU-P, but also the second isotope of GGC-P – over time. The experimental data plotted in c) and d) are from GC mixed polymerization at pH 11 after 1 dehydration of 24 hours.

Although it was a GC sample, it looks like we have some amount of GU product as well, due to the overlap between the isotopic distributions. This can be a problem, in particular of the AUGC mixed polymerization. Thus, quantification of the oligomers by integrating the area of the peak detected in the EIC chromatogram is not reliable in this case.

MS data analysis pipeline

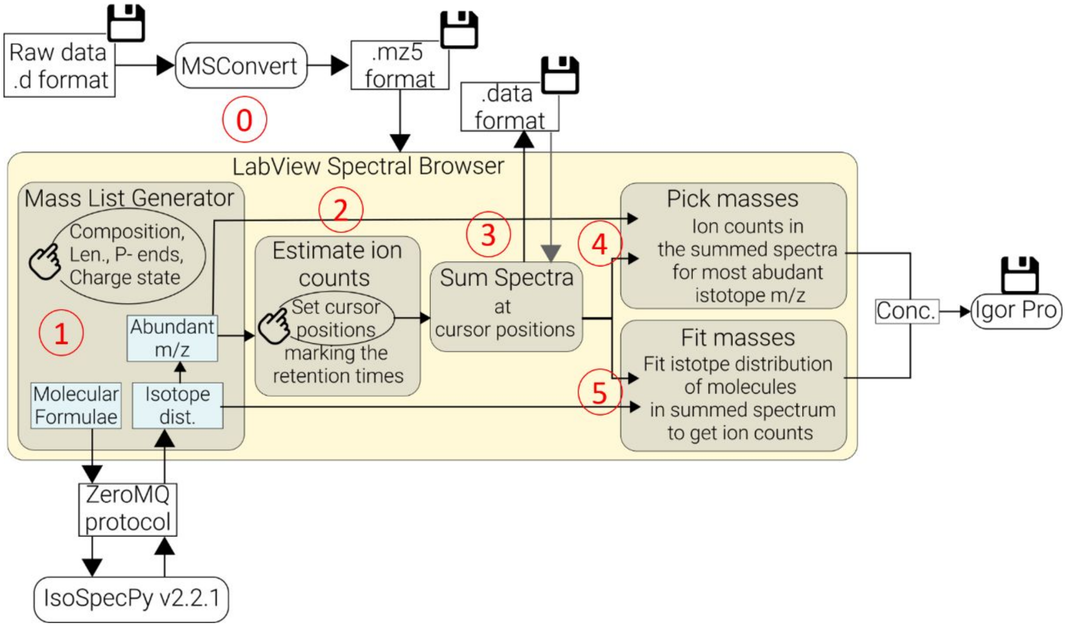

Figure S7. Schematic representation of the MS data analysis pipeline. The MS data analysis pipeline involves a step-by-step (0 to 5) process of data conversion (step 0), generating mass-list and theoretical isotope distributions (step 1), estimating ion counts over time to generate EIC chromatograms and set cursor positions marking the peaks (step 2), summing the raw spectra between the cursor positions (step 3), determining the ion counts either by the most abundant monoisotopic m/z (step 4: Pick masses) or by fitting the theoretical distribution (step 5: Fit masses). The total ion counts are then converted to concentration using the calibration coefficients and exported to Igor Pro for plotting and further analysis. The hand icon denotes the user input parameters, and the drive con shows the saved files.

Firstly the data obtained from the instrument – which is in a proprietary *.d* format – is converted to an open-source MS data format, *.mz5,* using MSConvert, a component of ProteoWizard [2]. Subsequently, it was imported into a custom LabVIEW program (Spectral_browser_3.58 or a newer version Spectral_browser_4.63) for detailed analysis (reported as Step 0 in Figure SM7). Version differences primarily involve additional analysis modalities for other MS datasets and bug fixes, but the core pipeline remains unchanged.

The workflow in the LabVIEW program consists of the following steps, also reported in Figure **S7**.

1. Mass List generation

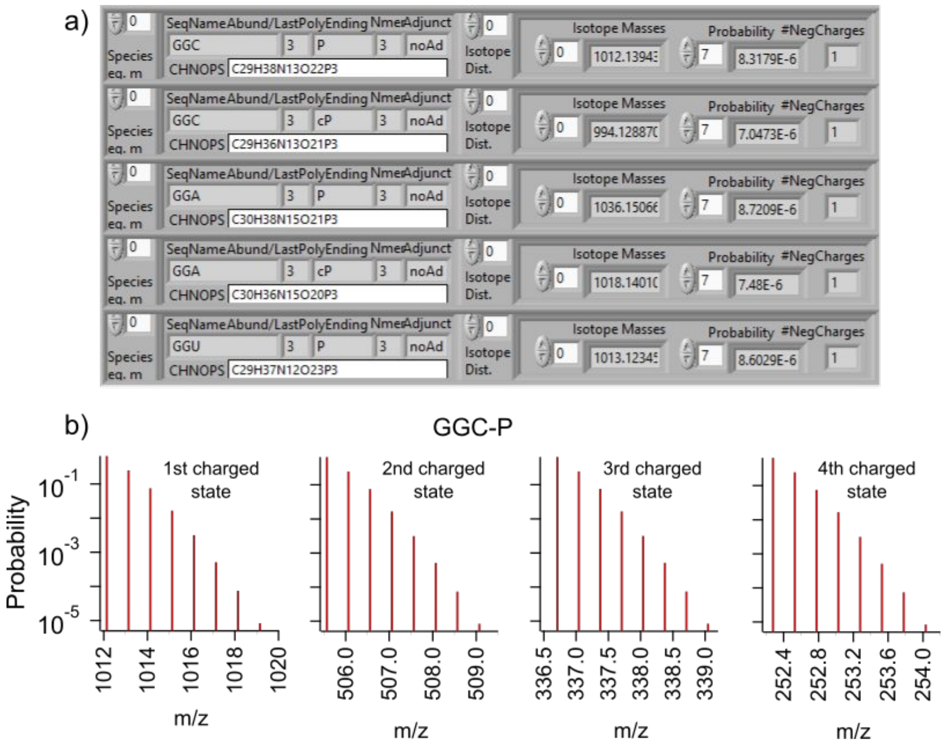

Figure SM8. Example of the mass list generated by the program for a AUGC mixed polymerization. a) Molecule list generated for oligomers of composition A/U/G/C, for lengths going from 1 to 3 nucleotides (on the Figure, you can only see some of the 3mers), with 2′/3′ ending either a cyclic (cP) or linear (P). b) For each molecule (here GGC-P), the mass list includes the 1st charged state (ionization of the molecule by removing 1 H) to the 4th charged state (removal of 4 H) and the 8 first isotopes of the molecule's isotopic distribution.

- This part of the program generates first the chemical formula for the different molecules to be analyzed. For oligomerization data, input parameters include the nucleotide composition (eg. A,U,G,C), length-range, (e.g. 1-5mers), possible phosphate end, (e.g. cyclic phosphate cP in Figure SM8) linear Phosphate P) and the charged states (e.g. 1-4)**,** to generate the different chemical formulae. The charge states needed depend on the length range of oligonucleotide and the ionization of the MS.
- These chemical formulae are then passed via ZeroMQ message transfer protocol (used for transferring data between different software components) to Python where IsoSpecPy[2] calculates the theoretical isotope distribution for the corresponding chemical formulae. This is then passed on back to the LabVIEW program (also via ZeroMQ).

2. Extracted Ion Counts (EIC) chromatogram plotting

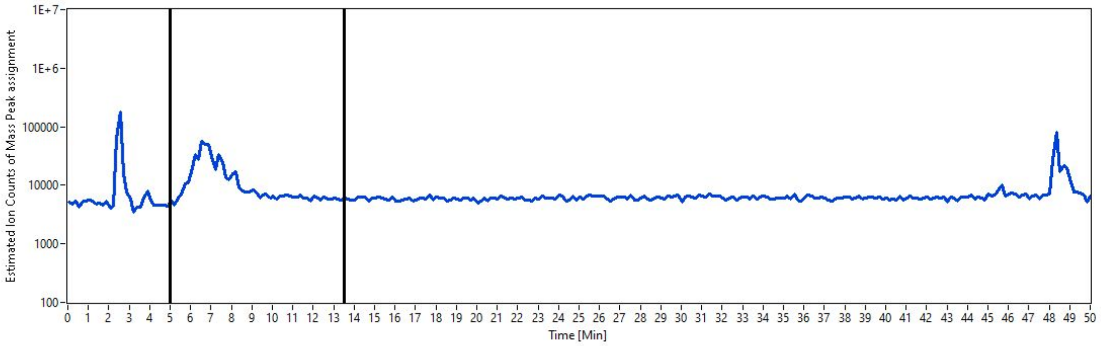

Figure S9. Example of EIC chromatogram for the oligomer product GGC-3'P plotted (in blue) for the most abundant isotope (for GGC-3'P, this corresponds to the first isotope) of the 1st charged state of the molecule (m/z = $[m_{GGC\text{-}3'P} - m_H]/1$ = 1012.139434) + 2nd charged state (m/z = $[m_{GGC\text{-}3'P} - 2*m_H]/2$ = 505.566074) + 3rd charged state (m/z = $[m_{GGC\text{-}3'P} - 3*m_H]/3$ = 336.708287) + 4th charged state (m/z = $[m_{GGC\text{-}3'P} - 4*m_H]/4$ = 252.279394). The two vertical black lines correspond to the cursors which delimit the scans that will be taken into account in step 3. The experimental data used there come from AUGC mixed polymerization at pH 10 after 10 wet-dry cycles.

- The m/z of the most abundant isotope for each molecule is plotted against time, generating extracted ion count chromatograms (EICs). It is important to note that these EICs are not used for quantifying (i.e by integrating the peak area) and only serve as visual guides for estimating retention times.
- Retention times are determined by comparing EICs of the sample to that of standard oligonucleotides (see section LC-MS calibration and analysis).
- The cursor positions marking the start and the end of the peaks are set manually (black vertical lines in Figure **S9** at 5 and 13.5 min).

<u>3. Summing of raw mass spectra at retention times</u>

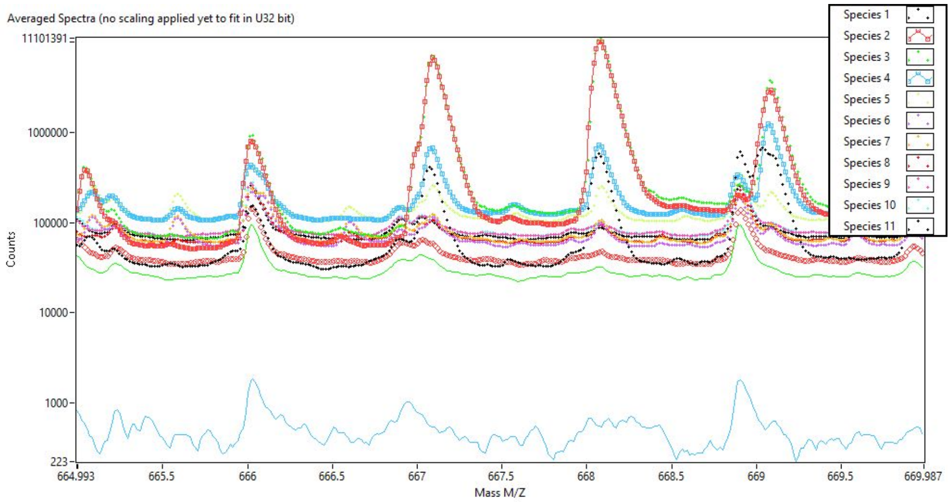

Figure S10. Summing of all spectra taken between the cursors positions: The LC-MS acquisition method was set so that 1 spectrum is taken every second. Here, all of the spectrum corresponding of retention times between 5 min and 13.5 min (see Figure **S10**) were added together to make a combined spectrum.

- Raw spectra between the cursor positions are summed to generate a combined spectrum for each retention time range (see Figure **S10**). Portions of that resulting combined spectrum can be seen in Figure **S11**: they correspond to the m/z values of the expected isotopic distributions of GGC-P.
- This relates each summed spectrum to a group of molecules that are expected at the corresponding retention time (in the case of the experimental data shown in Figure **S9**, the range of [5 ; 13.5] min corresponds to 4mer-cP and 3mer-P, e.g. GGC-P).

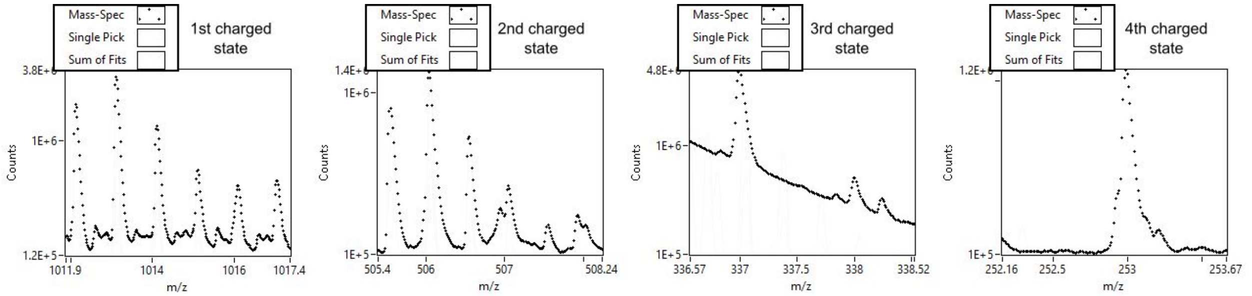

Figure S11. Portions of the spectrum resulting from the addition of all of the spectra taken between retention time of the two cursors in Figure **S5**. The four windows correspond to the m/z values of the isotopic distributions (1st to 4th charged states) of the product GGC-P. The black dots correspond to the experimental data obtained for a AUGC mixed polymerization at pH 10 after 10 wet-dry cycles.

4. Pick and 5. Fit of the summed isotope distribution and quantification.

- The quantification of the molecules in each summed spectrum can be done either by using the monoisotopic mass of the most abundant isotope (Pick), or by fitting the whole distribution (Fit).
- Pick: For each molecule, the theoretical isotopic distributions are aligned and scaled to the corresponding summed spectrum using the most abundant m/z as reference point (see Figure **S12** for the example of GGC-P). The scaling factor represents the measured ion counts for a given molecular charge state. The counts for each charge state are then summed to give the total ion counts.

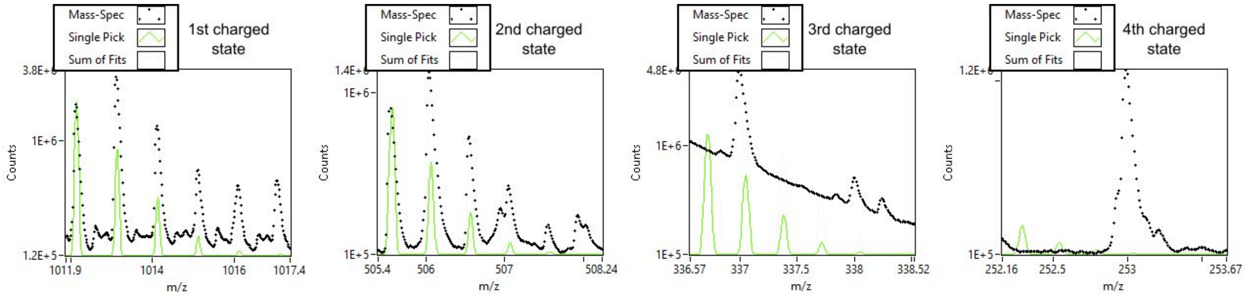

Figure S12. Pick step for the isotopic distributions of GGC-P. The black dots correspond to the experimental data (AUGC mixed polymerization at pH 10 after 10 wet-dry cycles), the green line to the

pick (the expected distributions for the GGC-P molecule, scaled up using only the abundance of the first isotope as a factor).
It needs to be noted that the molecule here is detected only in the first two charged states. The 3rd and 4th are just background noise and are not taken into account.
But even for the 1st and 2nd charged states, the experimental data do not follow the profile of the theoretical distribution here. This is due to overlaps with the isotopic distribution of the GGU-P molecule, which needs to be corrected in the next step.

- Fit: For molecules with overlapping isotope distributions, a combined theoretical isotope distribution of all molecules at a given cursor position is made. A subset of isotopes is selected and the combined theoretical distribution is fitted for each cursor position (in red in Figure **S13**). By default, three isotopes are used, as this balances the trade-off between accuracy and overfitting. Using too few isotopes can lead to inaccurate fits, while including too many low-abundance isotopes can cause overfitting or fitting failures. Similar to the case above, the total ion count is calculated by summing the counts for all charge states.

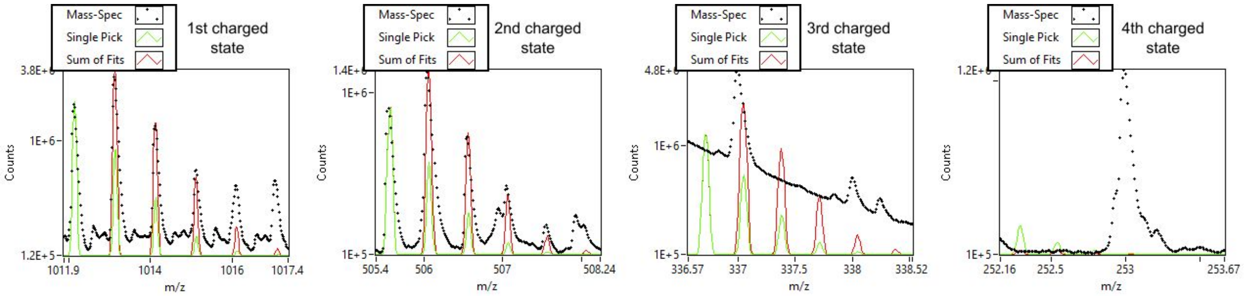

Figure S13. Fit step for the isotopic distributions of GGC-P. The black dots correspond to the experimental data (AUGC mixed polymerization at pH 10 after 10 wet-dry cycles), the green line to the pick (the expected distributions for the GGC-P molecule, scaled up using only the abundance of the first (most abundant) isotope as a factor) and the red line to the fit taking into account all of the potential overlaps with other molecules (here in particular GGU-P).
The calculated fit behaves similarly to the experimental data for the 1st and 2nd charged states.
The concentrations of GGC-P and GGU-P are corrected using that fit.

- Quantification coefficients are determined using standard oligonucleotides (see Polymers quantification section). These are then applied to relate these total ion counts to molar concentrations for the given sample volume.
- The calculated concentrations of all molecules are exported to Igor Pro for visualization and further analysis.

A complementary description of the Spectral_Browser program and the analysis method can be found in previously published work [1].

MS determination of reaction yield and products concentrations

The yields are calculated over the total concentration (in µM) of monomers A, U, C or/and G (remaining cyclic monomers + hydrolyzed monomers + monomers included in oligomers):

$$C_{tot} = \sum_{i=1}^{j} \sum_{N} i \times (C_{N_i-cP} + C_{N_i-P})$$

with N = {G, C, A, U}, i the length of the oligomer N_i, j the maximum length of oligomers, -cP indicating a cyclic ending, -P indicating a linear ending and C the concentration calculated by LC-MS quantification.

The polymerization yield for cyclic ending oligomers is defined as the total concentration of monomers included in an oligomer with a cyclic ending over the total concentration of monomers:

$$Y_{cP}^{tot} = \sum_{i=2}^{j} \sum_{N} i \times C_{N_i-cP} \div C_{tot}$$

The polymerization yield for linear ending oligomer is defined as the total concentration of monomers included in an oligomer with a linear ending over the total concentration of monomers:

$$Y_{P}^{tot} = \sum_{i=2}^{j} \sum_{N} i \times C_{N_i-P} \div C_{tot}$$

The total polymerization yield is defined as:

$$Y_{tot} = Y_{cP}^{tot} + Y_{P}^{tot}$$

The yield of hydrolysis of the monomers is defined as the concentration of linear-ending monomers over the total concentration of monomers:

$$Y_{P}^{1} = \sum_{N} C_{N-P} \div C_{tot}$$

The total concentration of oligomers of length *n*, define as C_{tot}^{n} is calculated by summing the concentration C_{Ni}^{n} of all the products of length *n* no matter their A/U/G/C composition.

The expected product distribution for random polymerization (RP) is calculated for each length *n* by dividing $C_{tot}^{n}$by the number of possible sequences/arrangements (e.g. for dimers in a AU sample there are 4 possible sequences: AA, UU, AU and UA) and multiplying it, for each combination (AA, UU and AU, as the LC-MS method cannot differentiate AU and UA), by its number of possible permutations (1 for AA, 1 for UU and 2 for AU). For example, the measured concentration of AA, UU and AU at cycle 10 and pH = 10 (Fig. 4a) are 775.5 µM, 307.6 µM and 1065.4 µM, respectively. Thus, the total concentration of dimers is 775.5 µM + 307.6 µM + 1065.4 µM = 2150.6 µM and the RP calculated concentrations are for AA and UU 2150.6 µM / 4 = 537.6 µM and for AU 2* 2150.6 µM / 4 = 1075.5 µM.

The rehydrated aliquots of the samples were injected into the LC-MS. The maximum detected length of products was dependent on the size of the aliquots. The concentrations of oligomers are normalized over a starting concentration of 50 mM.

Comparison between HPLC and LC-MS analyses

Each sample has been prepared in duplicates and the two experiments have been analyzed with both HPLC and HPLC-MS as described above and in the Methods section of the main text. In figures **S14** and **S15** we show the average between the data measured with the two different techniques (symbols), and the confidence level (shadings), calculated as the semi dispersion of the measures. Overall, all the samples are compatible with some variation due to experimental errors.

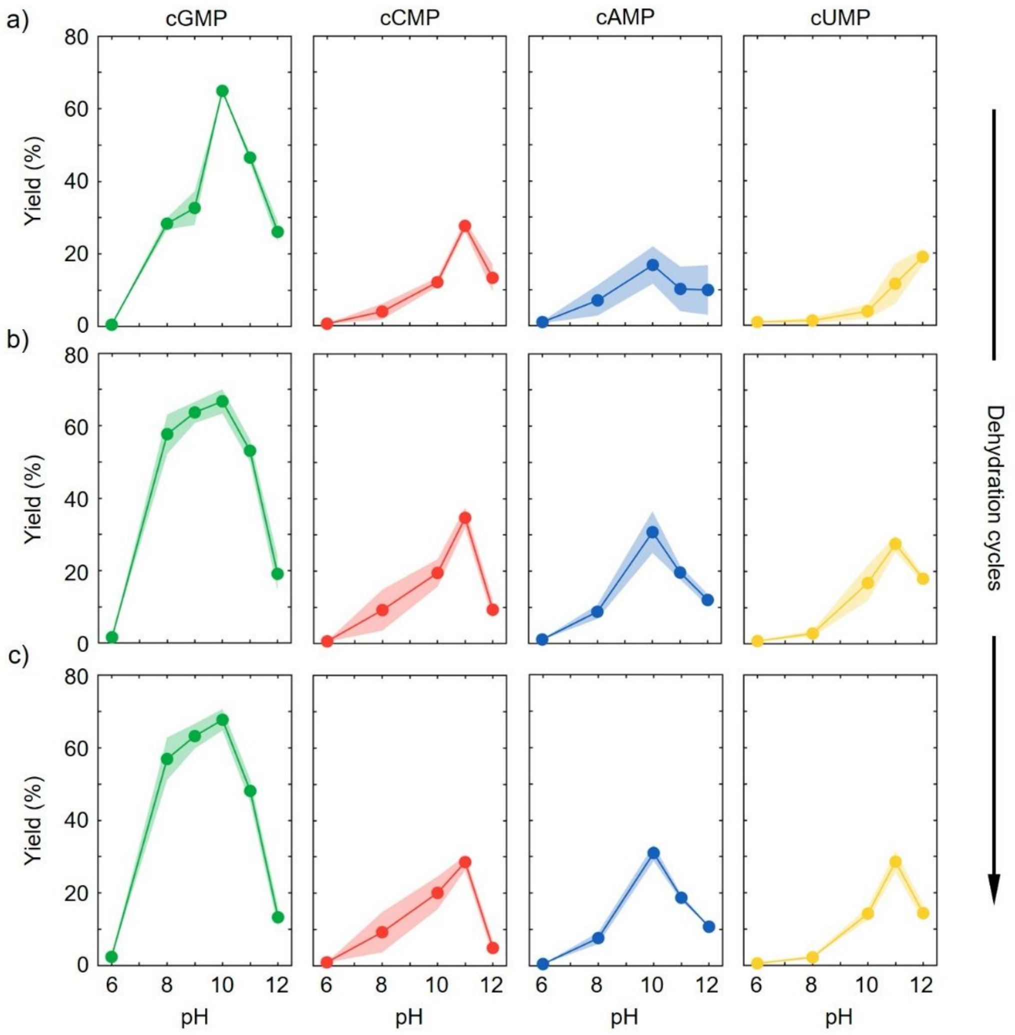

Figure S14. Comparison of the reaction yields of individual nucleotide solutions measured by HPLC and HPLC-MS. Yields for the polymerization of individual nucleotides (G, C, A, U) solutions as function of the initial pH and after n = 1 (**a**), n =5 (**b**) and n = 10 (**c**) dehydration-rehydration cycles at room temperature are calculated as the average between the HPLC and HPLC-MS analysis (see **methods**) and plotted as the average (symbols) and semi dispersion (light color shading).

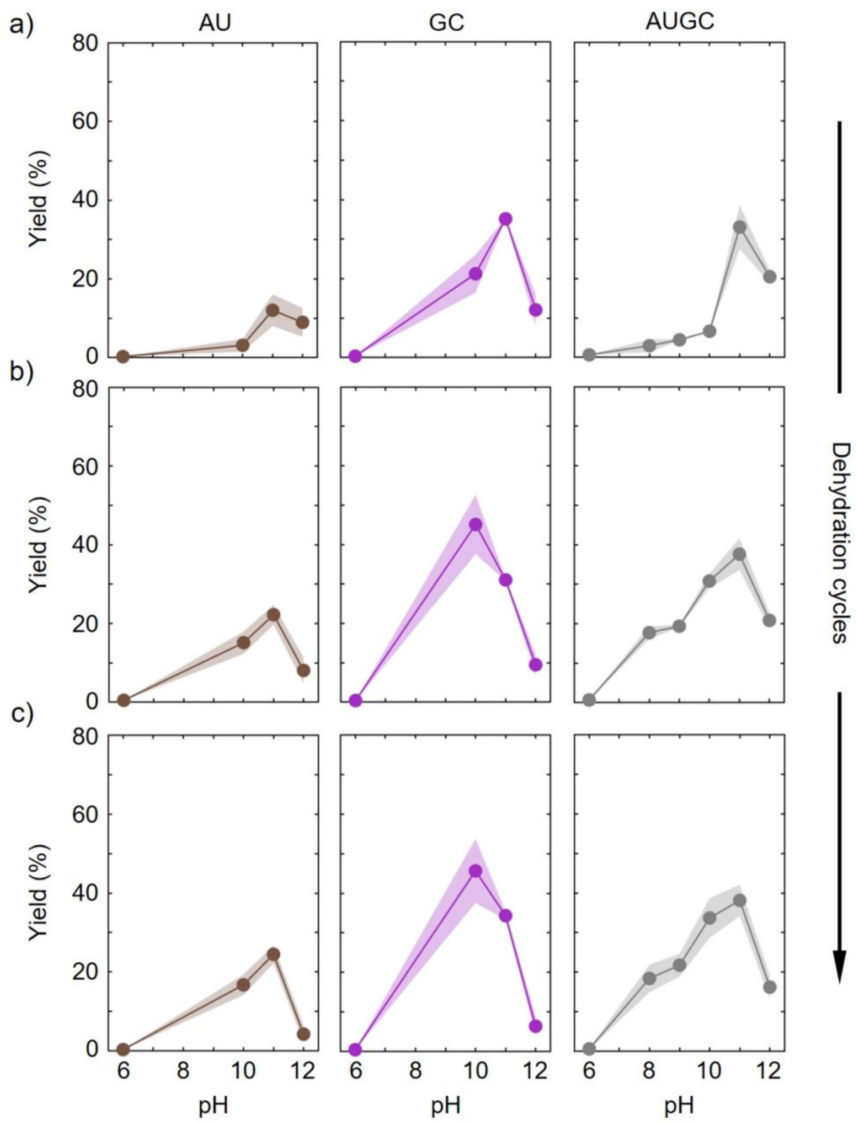

Figure S15. Comparison of the reaction yields of mixed nucleotide solutions measured by HPLC and HPLC-MS. Yields for the polymerization of nucleotide mixtures (AU – brown, GC – violet and AUGC – grey) solutions as function of the initial pH and after n = 1 (**a**), n =5 (**b**) and n = 10 (**c**) dehydration-rehydration cycles at room temperature are calculated as the average between the HPLC and HPLC-MS analysis (see **methods**) and plotted as the average (symbols) and semi dispersion (light color shading).

Supporting figures

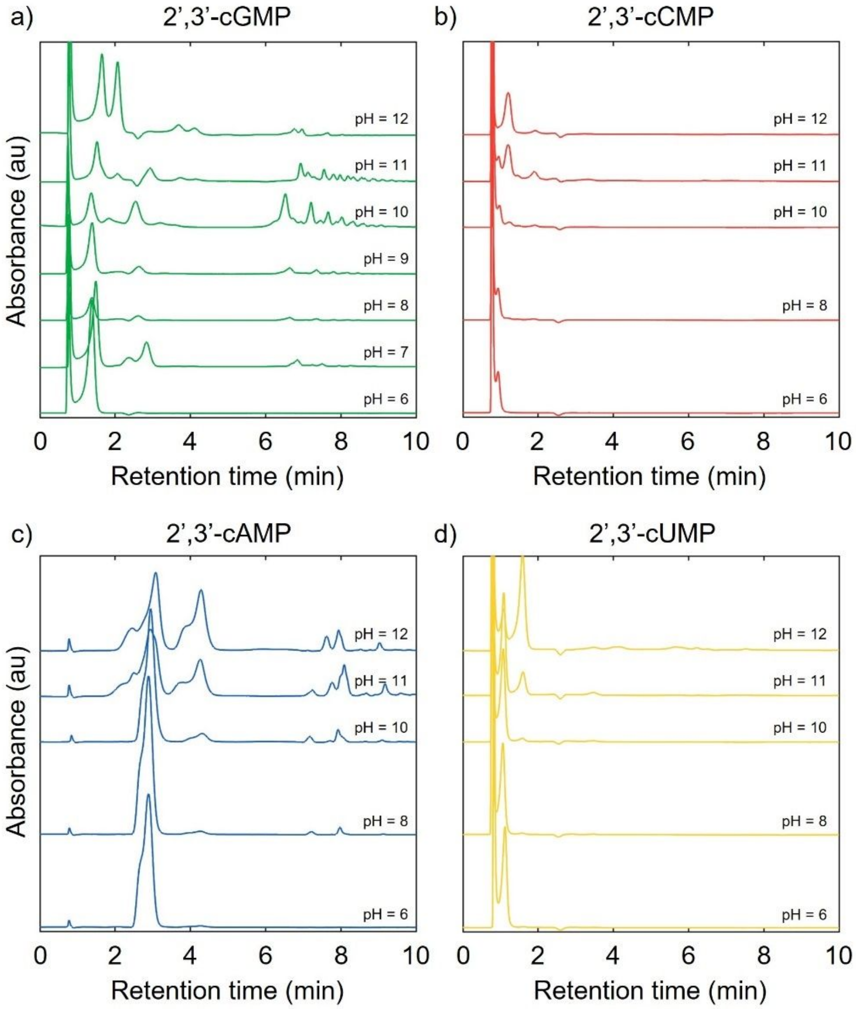

Figure S16. HPLC analysis of the oligomerization of individual solutions of 2',3'-cNMPs as a function of the initial pH. Traces of the HPLC absorbance at 260 nm of the dry state oligomerization of single nucleotide solutions of cGMP (a), cCMP (b), cAMP (c) and cUMP (d) at initial pH 6-12. pH was adjusted using potassium hydroxide (KOH). For each reaction 20 µL of 50 mM cNMPs were dried and incubated at room temperature for 24 hours.

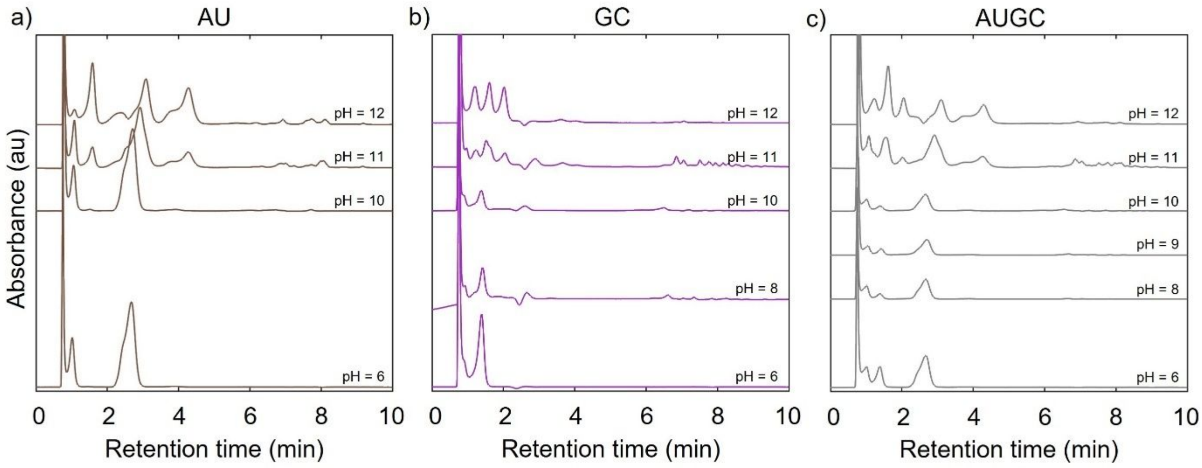

Figure S17. HPLC analysis of the oligomerization of binary and quaternary solutions of 2',3'-cNMPs as a function of the initial pH. Traces of the HPLC absorbance at 260 nm of the dry state oligomerization of binary mixtures AU (a) and GC (b); and quaternary mixture AUGC (c) at initial pH 6-12. pH was adjusted using potassium hydroxide (KOH). For each reaction 20 µL of 50 mM cNMPs were dried and incubated at room temperature for 24 hours.

a)

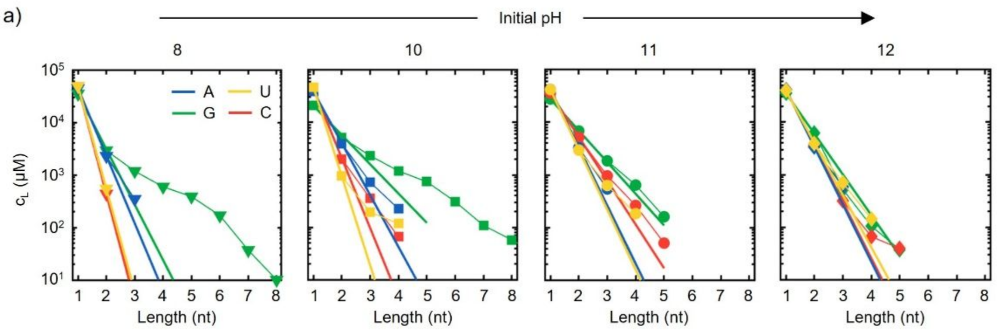

b)

Single exponential fit : $c_L = A \cdot e^{-\frac{L-1}{L_0}}$

pH = 8

	A (uM)	L_0 (nt)
A	4.44*10^4	0.34
U	4.89*10^4	0.22
G	3.50*10^4	0.41
C	4.91*10^4	0.21

pH = 10

	A (uM)	L_0 (nt)
A	3.90*10^4	0.44
U	4.71*10^4	0.26
G	7.49*10^4	0.78
C	4.46*10^4	0.32

pH = 11

	A (uM)	L_0 (nt)
A	4.18*10^4	0.39
U	4.15*10^4	0.38
G	2.75*10^4	0.73
C	3.5510^4	0.52

pH = 12

	A (uM)	L_0 (nt)
A	4.16*10^4	0.4
U	3.94*10^4	0.44
G	3.55*10^4	0.56
C	4.15*10^4	0.41

c)

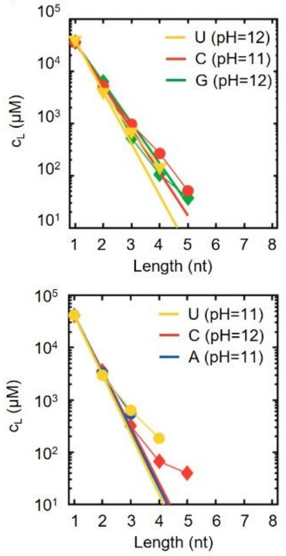

Figure S18. Products distribution as function of the pH. Oligonucleotide length (mole fraction) distributions produced by the drying of 2’,3’-cNMPs (c_0 = 50 mM, t = 24 hours), as a function of the pH. The fit with the exponential function $c_L = A*\exp(-(L-1)/L_0)$ (tick lines) reveals that the length distribution is not exponential, with the presence of higher concentration of oligonucleotides longer than the 2-mers (L > 2).

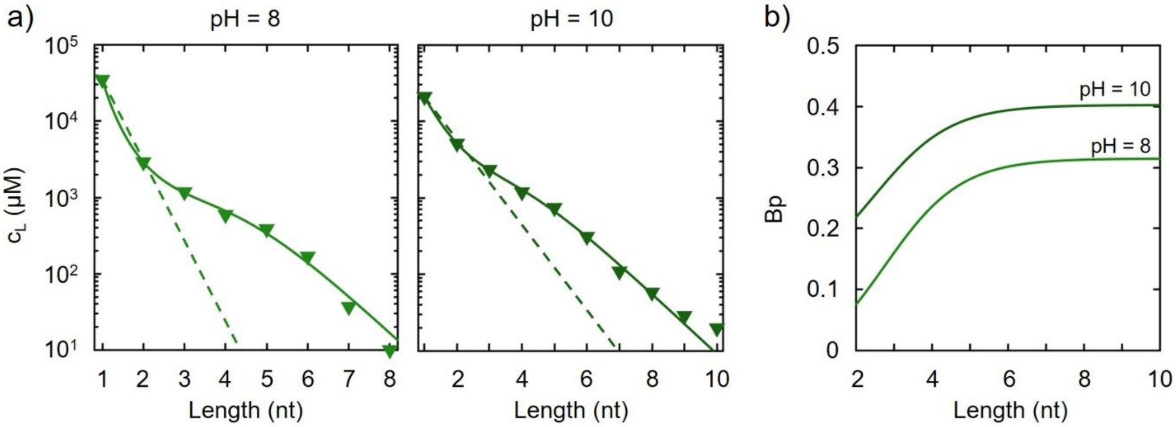

Figure S19. Products distributions of G at pH = 8 and 10 show length dependent reactivity. **a)** Exponential fit of products distributions at pH =8 (left) and pH=10 (right). The fitting equation is c_L=k·Bp^(L-1)·(1-Bp)^L, where Bp is the binding probability and k is a scaling constant to convert the probability in a concentration. Dashed lines: exponential fit with length independent binding probability (Bp = Bp_0). Solid lines: fit with a sigmoidal length dependent binding probability Bp = A_0+A/(1+exp(-(L-L_0))). **b)** Sigmoidal length-dependent binding probability Bp. Parameters are obtained from the fit in panel a.

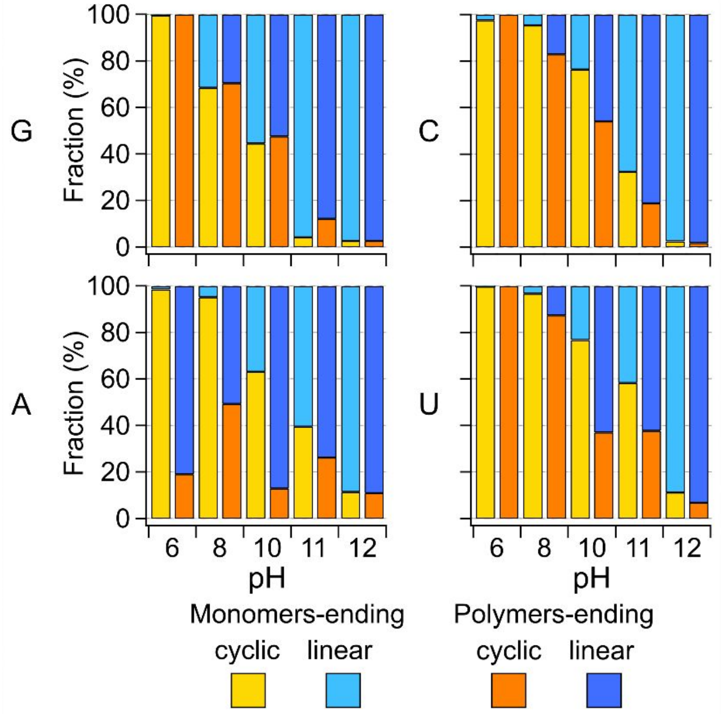

Figure S20. Fractions of 2',3'-cyclic and 2'/3'-linear phosphate endings for monomers and polymers after one dehydration cycle. The fractions were calculated based on the concentrations of monomers and oligomers of different endings measured by LC-MS analysis, t = 24h, for different starting pH, for each 2',3'-cNMPs (50 mM initial concentration). Data show that 2',3'-cyclic phosphate hydrolysis increases with rising pH, indicating that attack by water molecules or 5'-hydroxyl groups on the 2',3'-cyclic phosphate is both a necessary and limiting step of the reaction. The presence of a substantial amount of reactive 2',3'-cyclic phosphate termini after a 24-hour reaction suggests that polymerization can proceed in subsequent hydration-dehydration cycles.

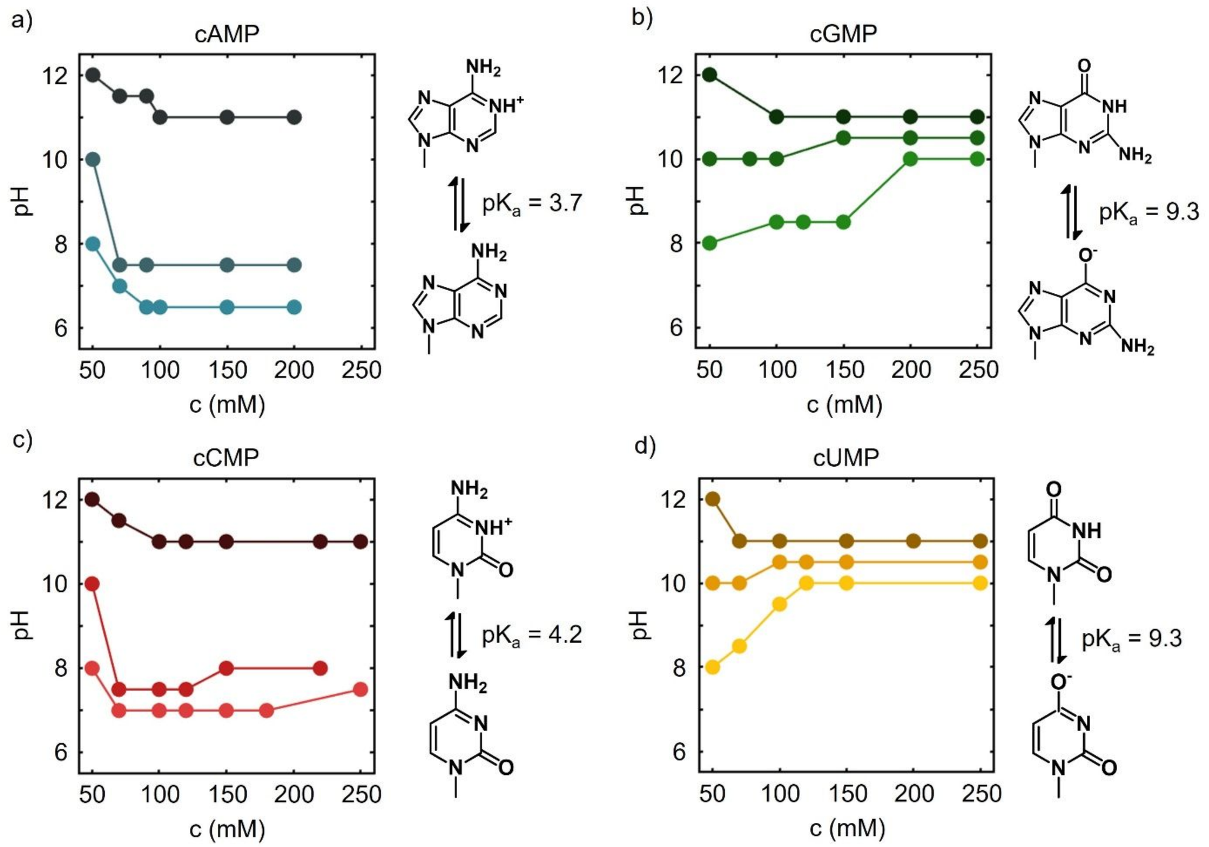

Figure S21. Variation of the pH of individual solutions of 2',3'-cNMPs during the evaporation process and drawing of the deprotonation of the nucleobases and their pka values. Plot of the pH values of 20 µL individual solutions of cNMP (50 mM), prepared at different initial pH (8-12), measured at different times during the evaporation process in plastic tubes for cAMP (a), cGMP (b), cCMP (c) and cUMP (d). Concentrations are calculated from the measurement of the mass loss of the solution during time. The pH drops can be attributed to acidification due to dissolution of CO_2 from the environment. The pH stabilization at alkaline pH of cGMP and cUMP is conceivably caused by the buffering effect of their nitrogen group (N1 in G and N3 in U) with pKa values of 9.3, contrary to cAMP and cCMP which have N1 with a pka of 3.7 and N3 with a pka of 4.2, respectively.

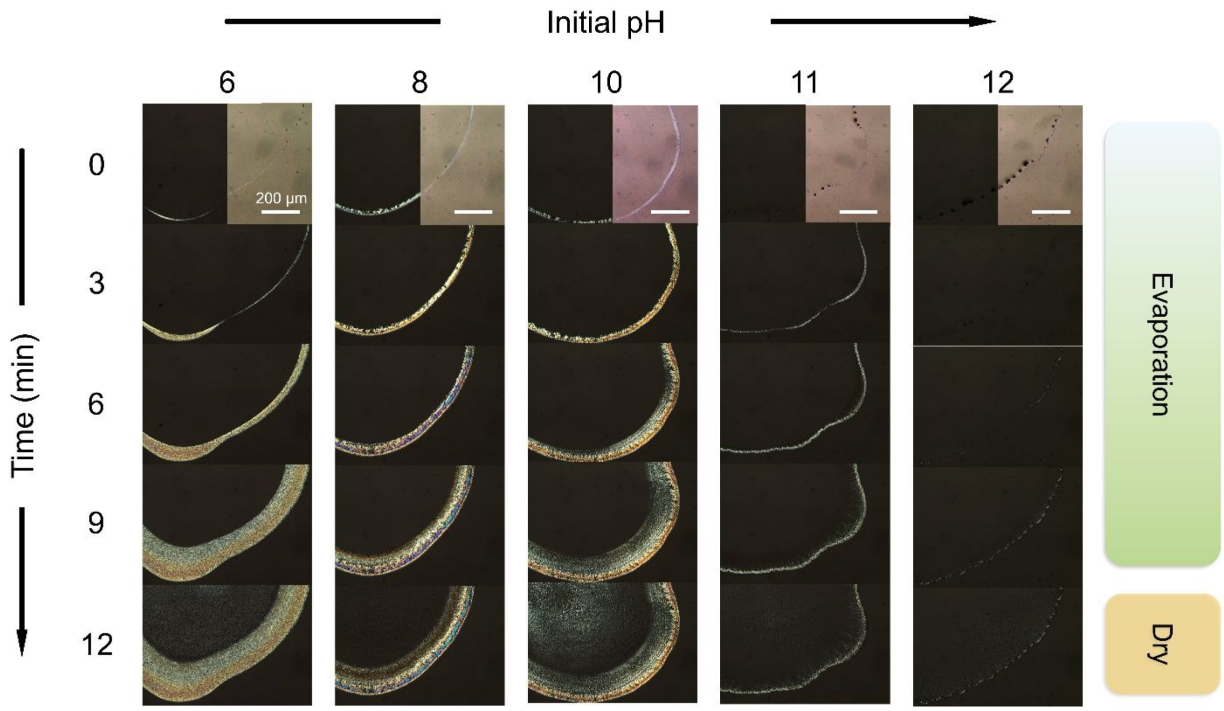

Figure S22. Liquid crystal and crystal assembly during cGMP drying dependence on pH. PTOM time lapse images of the evaporation of a 2 µL droplet of 50 mM cGMP solutions at different initial pH values (obtained with KOH addition). The extension of the birefringent regions, indicating liquid crystal and crystal domains, decreases at increasing pH, indicating the destabilization of G-quartets based self-assembly and ordering process. Images at t = 0 min are obtained by collated crossed polarized (left) and bright field (right) pictures. Scalebar is 200 µm.

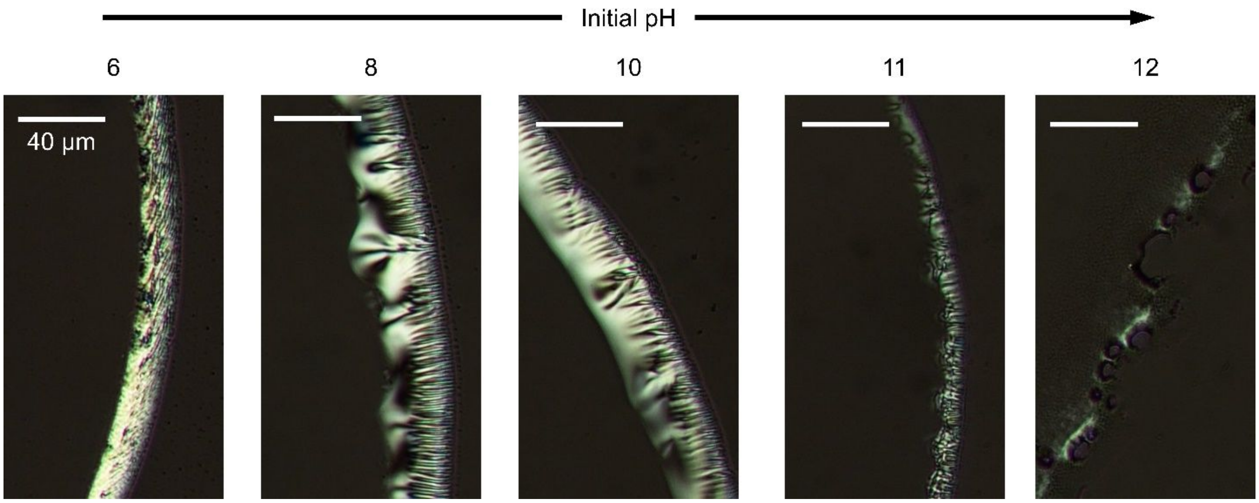

Figure S23. Details of liquid crystal domains as function of pH. Higher magnification images of LC regions growing at the border of the droplet during the evaporation, after 1 min, where local concentration increase is caused by "coffee ring effect" [3]. The decrease of the extension of the LC domains at increasing pH suggests that a destabilization of G-quadruplexes based self-assembly and LC ordering process is taking place.

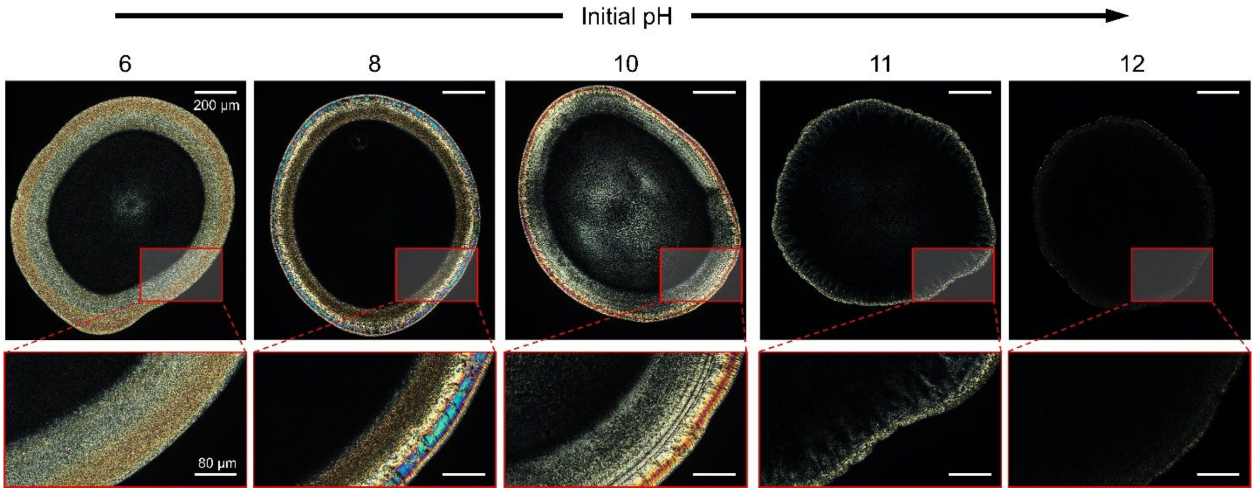

Figure S24. Dry state of cGMP samples at different initial pH. PTOM images of the full droplets and border details of the final dry state obtained after complete drying, t = 12 min, of cGMP solutions at 50 mM initial nucleotide concentration and initial pH = 6 – 12. Scale bars are 200 µm and 80 µm, respectively.

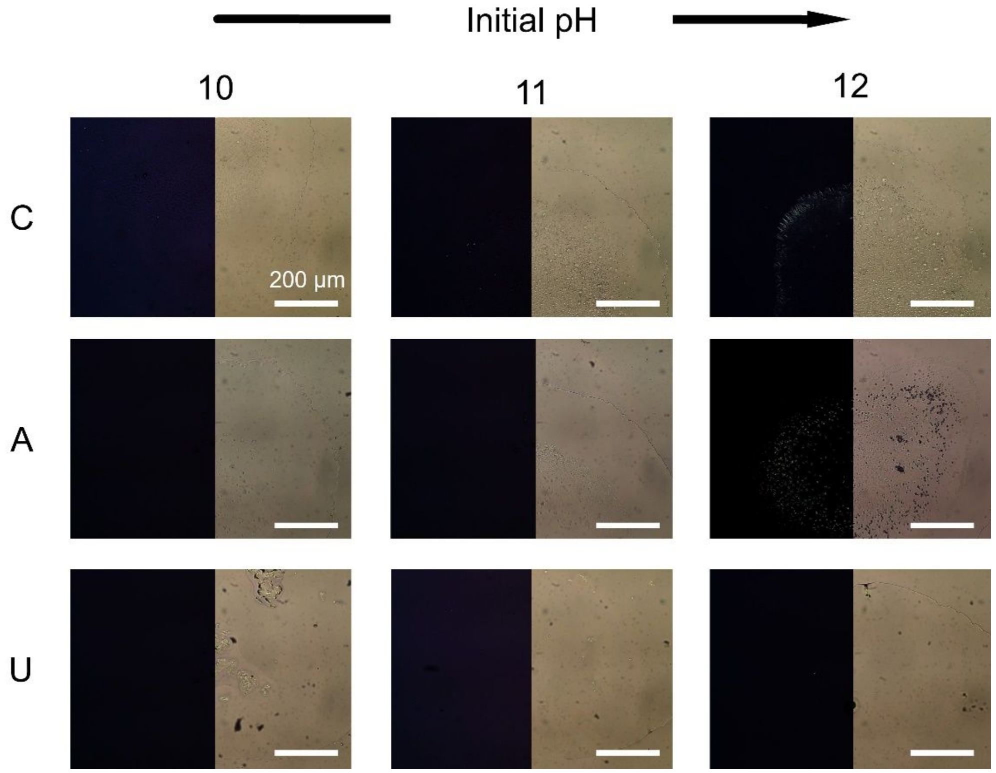

Figure S25. PTOM analysis of the dry state of A, U, C systems reveals unstructured isotropic state. PTOM images of the dry state of A, U, C prepared at 50 mM initial nucleotide concentration and different initial pH (10 – 12), taken with crossed polarizers (left), reveal the absence of birefringent crystalline domains comparable to those observed in presence of G (**Fig. S23**). Only KOH crystallization at pH = 12 is observed. Images taken without analyzer (right) show the dry state to appear as an amorphous glassy phase with inclusions of non-birefringent precipitate structures.

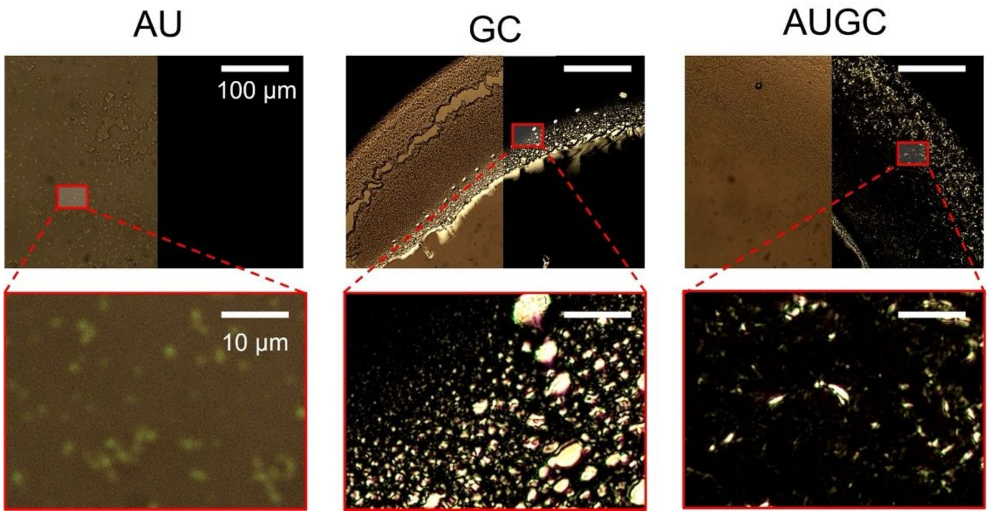

Figure S26. PTOM analysis of the dry state of AU, GC and AUGC mixtures. Top row: PTOM images of the first dehydration of AU, GC and AUGC mixtures prepared at 50 mM initial nucleotide concentration and initial pH = 10, in bright field (left) and crossed polarizers (right), reveal the presence of birefringent crystalline domains (white and colored regions) in coexistence with an isotropic glassy phase (dark regions) only for G-containing mixtures (GC and AUGC). In AU mixtures only an isotropic glassy phase is observed with the inclusion of non-birefringent precipitates. Bottom row: higher magnification images of the regions delimited by the red rectangles.

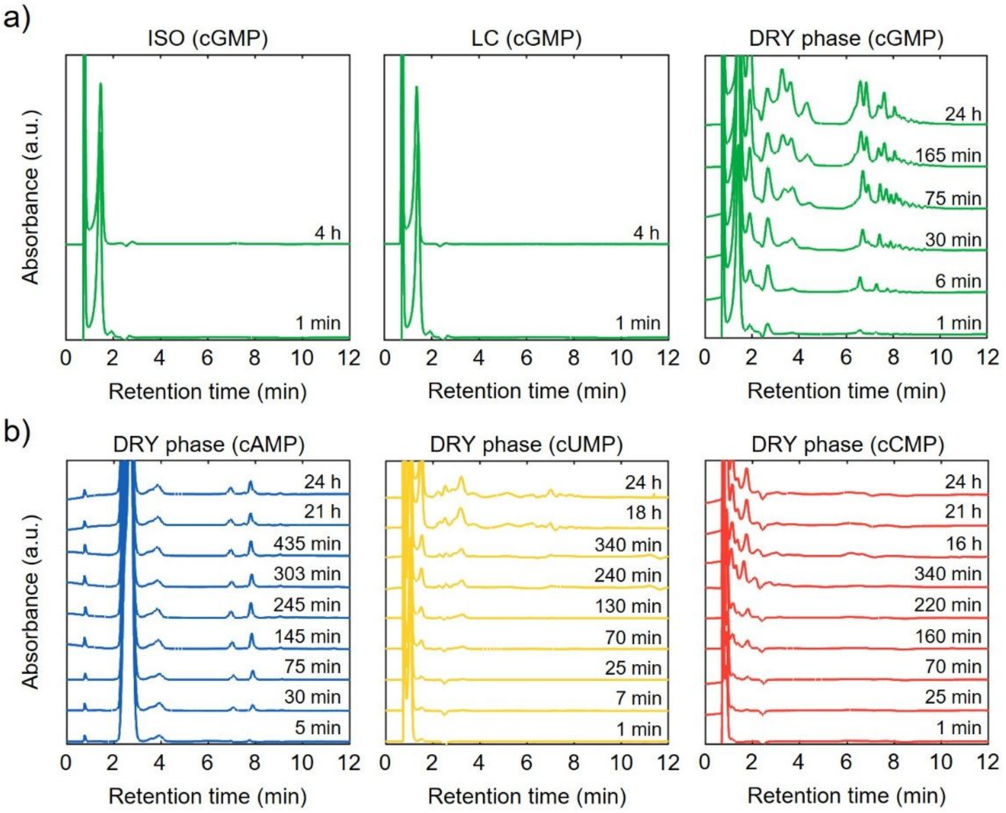

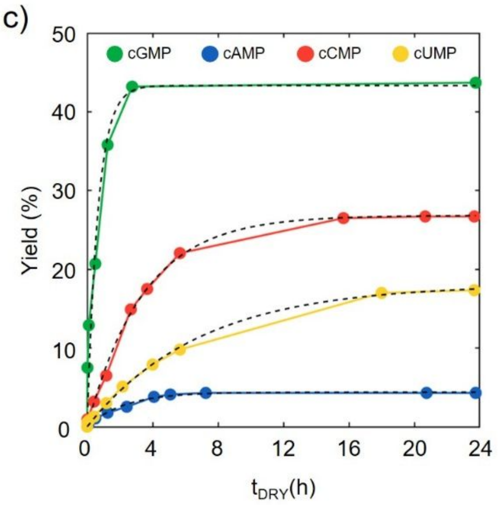

d)

$$yield = A \cdot (1 - e^{-\frac{t}{\tau}})$$

	A (%)	τ (h)
A	4.39	2.83
U	18.08	6.89
G	43.33	0.66
C	26.84	3.45

Figure S27. Reaction kinetics for the oligomerization of 2’,3’-cNMPs at pH = 11. a) Traces of the HPLC absorbance at 260 nm of the oligomerization of cGMP incubated in concentrated isotropic phase (left), in liquid crystal phase (center) and in dry phase (left) for increasing time. Absence of peaks at higher retention times indicate that the reaction is ineffective in ISO and LC phase. **b)** Traces of the HPLC absorbance at 260 nm of the oligomerization of cAMP (left), cUMP (center) and cCMP (right) incubated in dry phase (left) for increasing time. **c)** Plot of the oligomerization yield as a function of the incubation time in the dry phase measured from the HPLC traces for the different cNMP systems. Data were fitted with an exponential function y = A*(1-exp(-x/τ)) (dashed lines). **d)** Plot of the characteristic time τ of the oligomerization reaction obtained from the fit for the different cNMP.

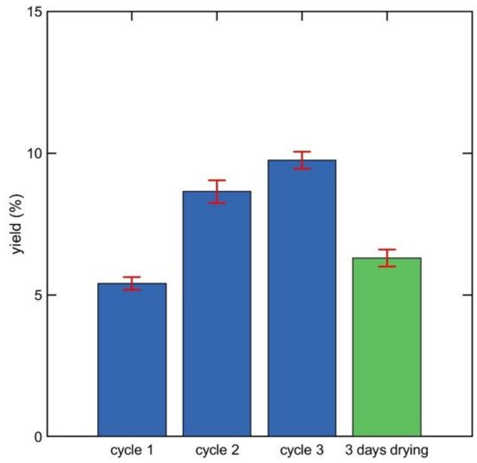

Figure S28. Comparison of the oligomerization of 2’,3’-cAMPs by dry-wet cycles and continuous drying. Reaction yields for the polymerization of 2’,3’-cAMPs measured by HPLC analysis after 1, 2 and 3 dry-wet cycles of 24 hours each (blue bars) and after 3 days in which the samples kept in dry conditions (green bar). Significant increase of the polymerization yield was observed when the samples were periodically rehydrated and dehydrated, while 3 days incubation in continuous dry condition caused only marginal increase of the reactivity. Experiments were performed in parallel and in three replicas. Data are reported as mean and standard deviation of three independent replicate.

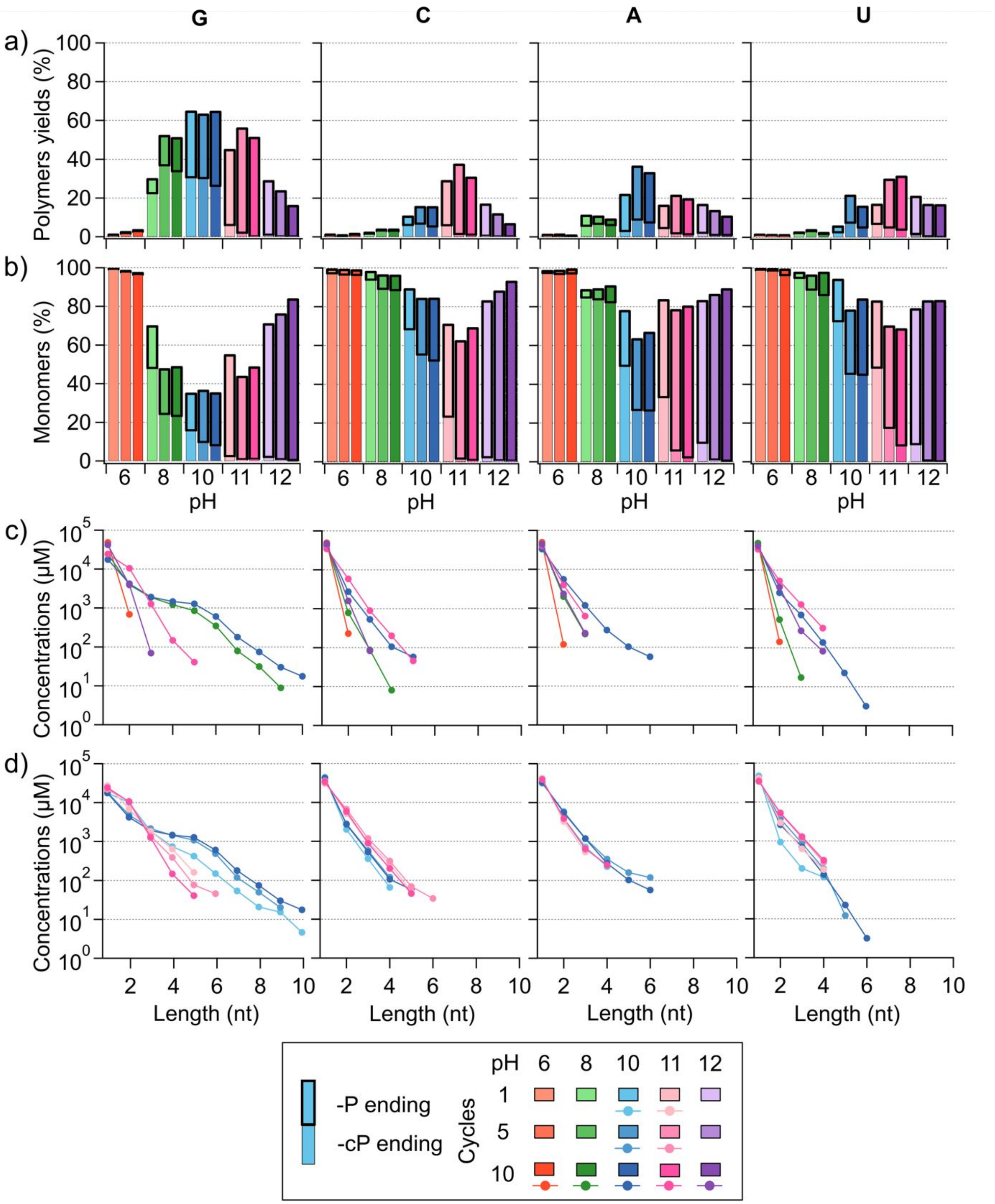

Figure S29. LC-MS analysis of individual cNMPs solutions after 1, 5 and 10 evaporation-rehydration cycles. a) Polymerization yields separating the cyclic-end and linear ending oligomers. b) Yields of remaining monomers, either with the reactive 2′,3′-cyclic phosphate end or with the hydrolyzed 2′- or 3′-phosphate end. c) Products length distribution for a starting pH 6, 8, 10, 11 and 12 after 10 cycles. d) Products length distribution for a starting pH 10, 11 and 1, 5 and 10 cycles.

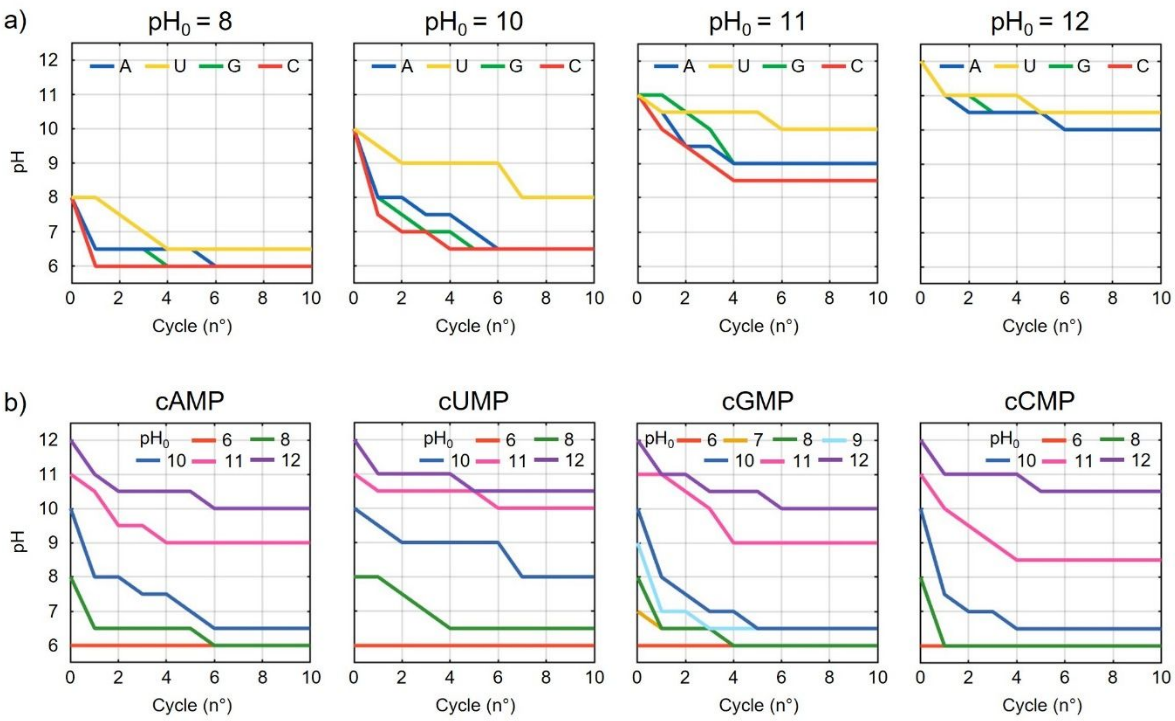

Figure S30. Measurement of pH drop of individual cNMPs solutions at each evaporation-rehydration cycle. a) Measured pH as function of the cycle number, n, for the different cNMP (G = green, C = blue, A = red, U = yellow) solutions at initial pH = 12, 11, 10 and 8. **b)** Measured pH as function of the cycle number, n, at different initial pH values (12 = violet, 11 = pink, 10 = blue, 8 = green, 6 = red) for each cNMP system. pH measurements were performed at each cycle (t = 24 h) after water addition and before subsequent evaporation. The pH drop can be attributed to the solution acidification due to dissolution of CO_2 from the environment, since no pH variation was observed when the process was performed under nitrogen atmosphere. In contrast to the pH variations observed during the first evaporation step (Fig. S6), no pH stabilization was detected for cGMP and cUMP during cyclical rehydration. This lack of stabilization is likely due to the diminished buffering capacity of their alkaline pKa under low-concentration conditions.

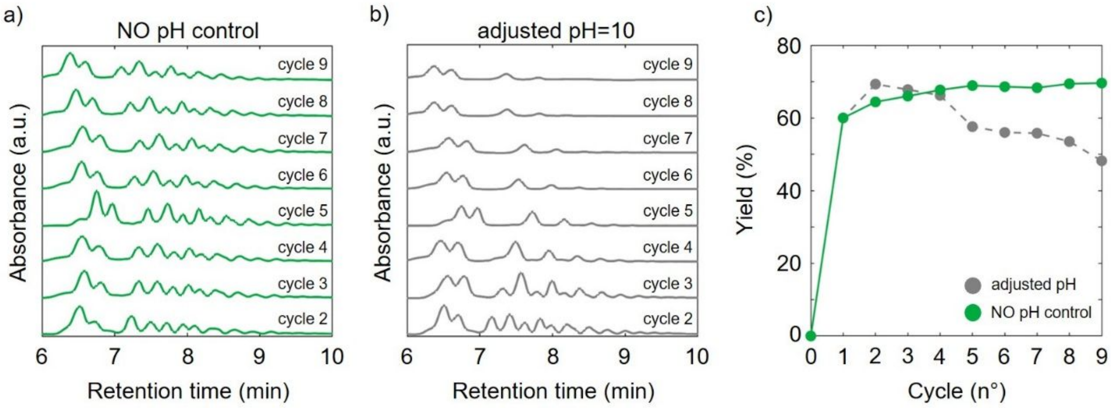

Figure S31. Comparison of the oligomerization of cGMP during dehydration-rehydration cycles with and without pH adjustment. Traces of the HPLC absorbance at 260 nm of the oligomerization of cGMP at each cycle when rehydration is performed by the addition of just water without pH control **(a)** or by the addition of KOH to adjust pH to 10 **(b)**. For both systems the initial pH was 10. **c)** Yield of the oligomerization of cGMP as function of the cycle number with (grey dots) and without (green dots) pH adjustment measured from the HPLC traces. The more rapid increase and subsequent decrease of the yield for adjusted pH = 10 with respect to unbuffered samples, indicates that the, as expected, high pH initially favors the opening of the 3',2'-cyclic phosphates (which speeds up the reactivity), but as the substrate is quickly consumed and the phosphodiester bonds start to be hydrolyzed (decreasing the amount and length of polymeric products).

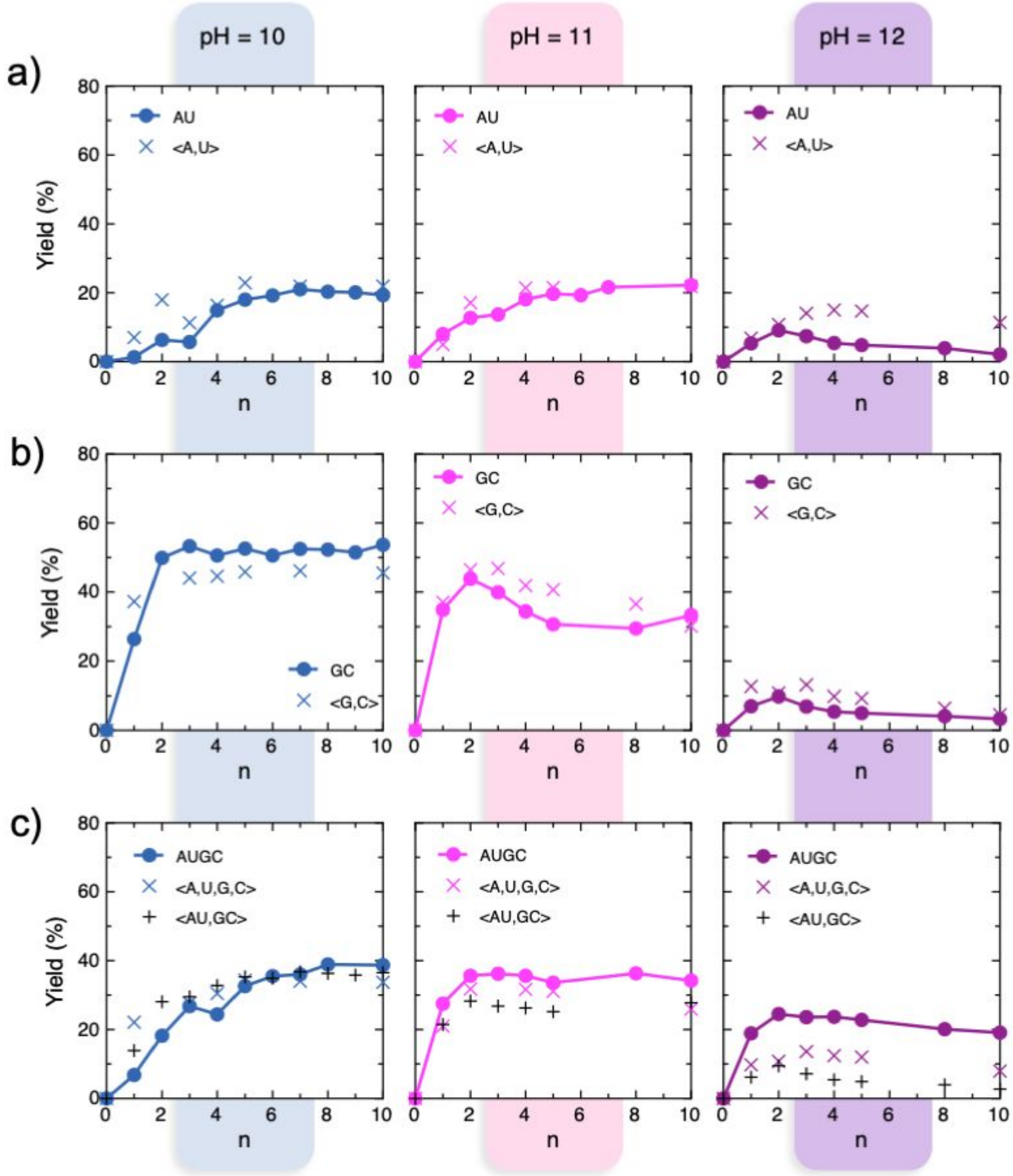

Figure S32. Comparison of the polymerization yield of mixtures and of single nucleotide systems. Polymerization yields, as a function of the number of cycles for AU (a), GC (b) and AUGC (c) mixtures at pH=10 (left), pH=11 (center) and pH=12 (right), are plotted as filled dots, and compared with the average of the polymerization yields measured for the relative single nucleotides systems, <A, U>, <G, C> and <A,U,G,C> respectively (x symbols). In panel c, AUGC yields are compared also with the average of the polymerization yields measured for AU and GC mixtures. Yield values were obtained from HPLC analysis (Fig. 3 of the main text and methods).

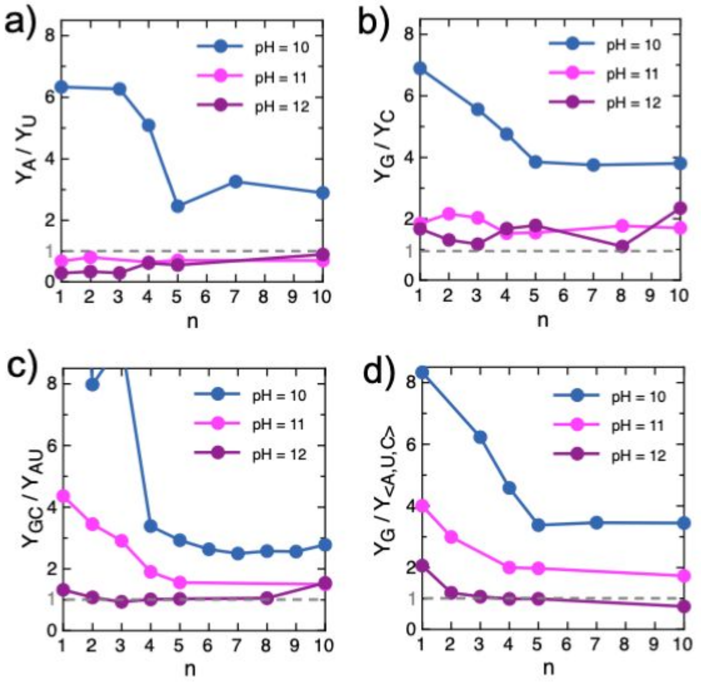

Figure S33. Ratios of the polymerization yields as function of the number of cycles (n) and initial pH. The ratios of polymerization yields measured for single A and U solutions (a), single G and C solutions (b), GC and AU binary mixtures (c), and single G solution and the average of single A, U, C solutions (<A, U, C>) are plotted as a function of the number of cycles and pf the initial pH (color code and legend). Grey dashed lines indicate the ratio = 1 (equal yields). For all the reported comparisons, the yields ratio decreases for pH ≥ 11 and for increasing n, suggesting that in such conditions heterogeneous polymerization may be enhanced in nucleotide mixtures. Yield values were obtained from HPLC analysis (Fig. 3 of the main text and methods).

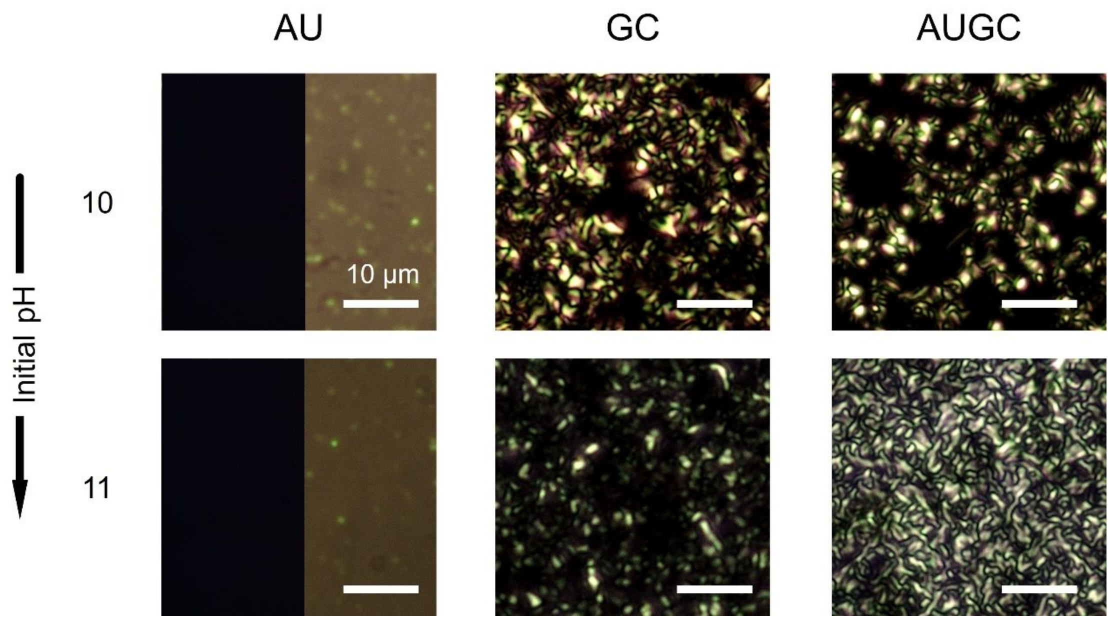

Figure S34. Comparison between the dry state of cNMPs mixtures. PTOM images of the dry state of AU, GC and AUGC after 6 dehydration cycles with initial pH = 10 and 11. Isotropic glassy single phase was observed for AU mixtures at initial pH = 10 - 11 (left side dark pictures, taken with crossed polarizers, show no presence of ordered structures but only micron size non-birefringent precipitates). Phase separations between a crystalline phase (bright birefringent domains) and an isotropic glassy phase (dark regions) were observed for GC at initial pH = 10 – 11 and AUGC at initial pH = 10. The dry state of AUGC mixture at initial pH = 11 instead showed a more homogeneous crystalline phase throughout the whole sample. Scale bars are 10 μm.

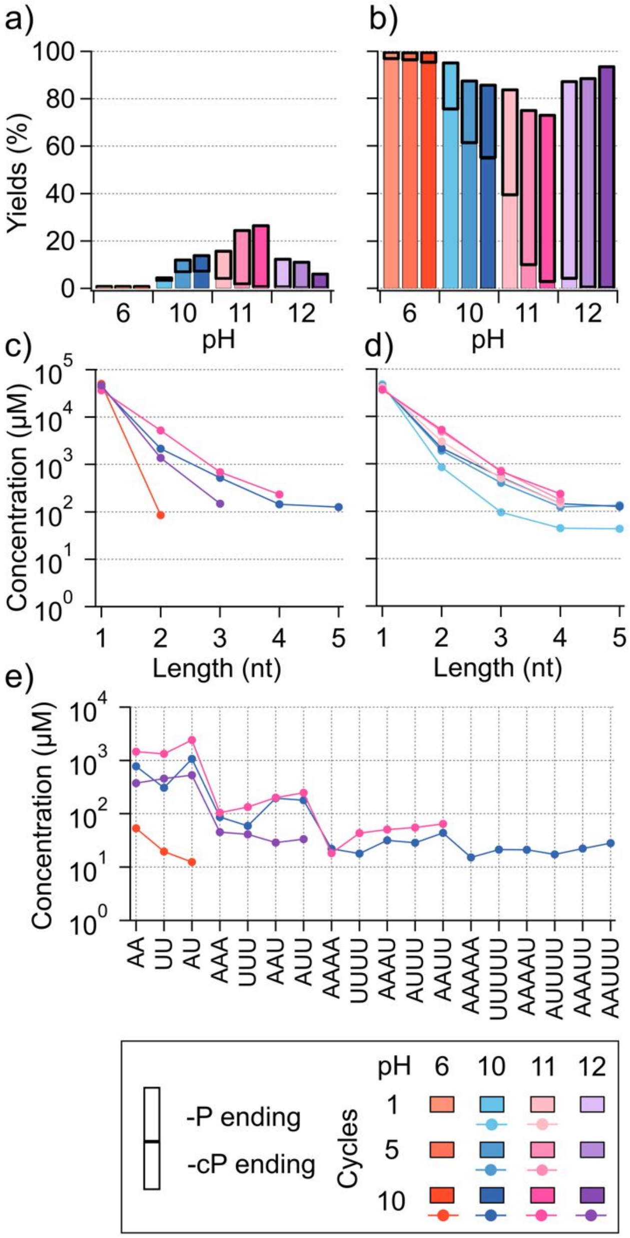

Figure S35. LC-MS analysis of the AU mixed samples. a) Polymerization yields separating the cyclic-end and linear ending oligomers. b) Yields of remaining monomers, either with the reactive 2′,3′-cyclic phosphate end or with the hydrolyzed 2′- or 3′-phosphate ends. c) Products length distribution for a starting pH 6, 8, 10, 11 and 12 after 10 cycles. d) Products length distribution for a starting pH 10, 11 and 1, 5 and 10 cycles. e) Detail of the product composition distribution after 10 cycles for a starting pH 6, 10, 11 and 12 for lengths of 2 to 5-mers.

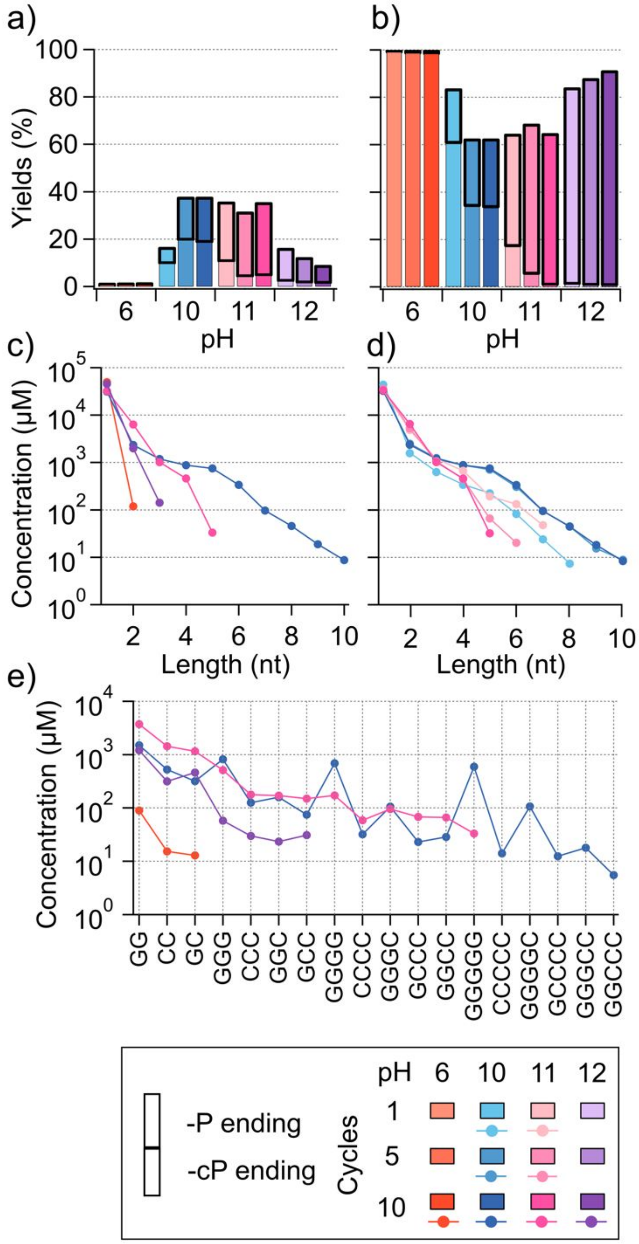

Figure S36. LC-MS analysis of the GC mixed samples. a) Polymerization yields separating the cyclic-end and linear ending oligomers. b) Yields of remaining monomers, either with the reactive 2′,3′-cyclic phosphate end or with the hydrolyzed 2′- or 3′-phosphate ends. c) Products length distribution for a starting pH 6, 8, 10, 11 and 12 after 10 cycles. d) Products length distribution for a starting pH 10, 11 and 1, 5 and 10 cycles. e) Detail of the product composition distribution after 10 cycles for a starting pH 6, 10, 11 and 12 for lengths of 2 to 5-mers.

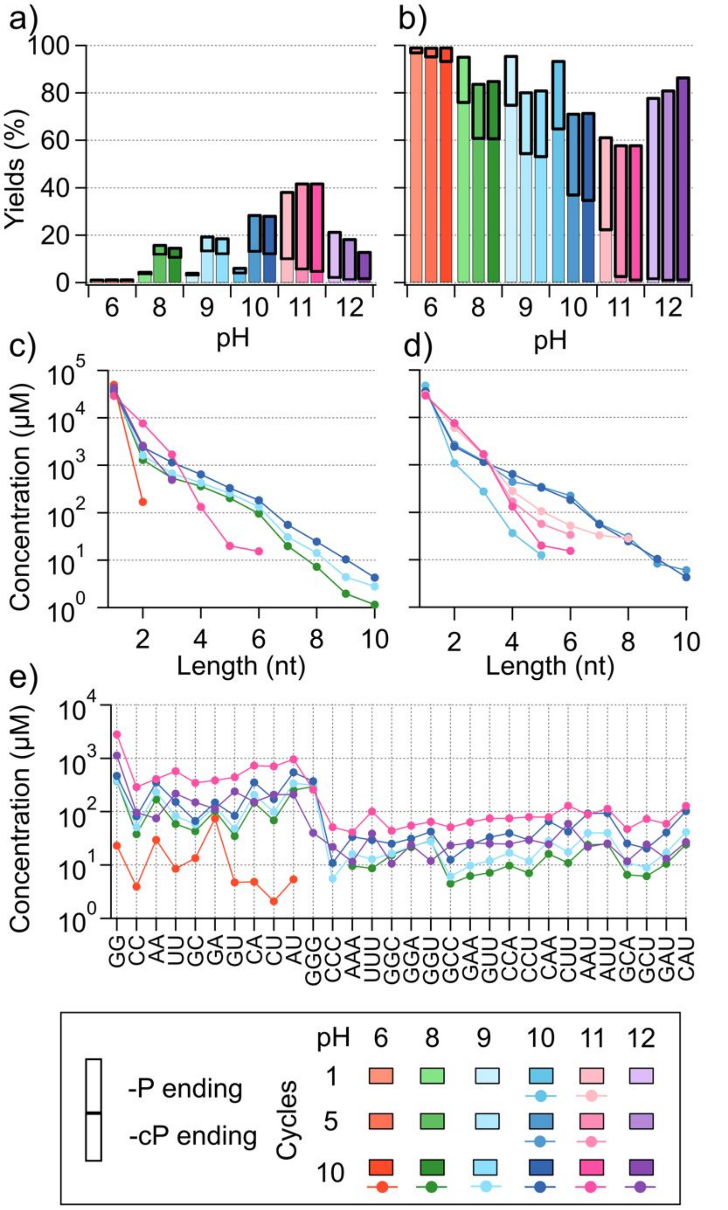

Figure S37. LC-MS analysis of the AUGC mixed samples. a) Polymerization yields separating the cyclic-end and linear ending oligomers. b) Yields of remaining monomers, either with the reactive 2′,3′-cyclic phosphate end or with the hydrolyzed 2′- or 3′-phosphate ends. c) Products length distribution for a starting pH 6, 8, 10, 11 and 12 after 10 cycles. d) Products length distribution for a starting pH 10, 11 and 1, 5 and 10 cycles. e) Detail of the product composition distribution after 10 cycles for a starting pH 6, 10, 11 and 12 for lengths of 2 to 3-mers.

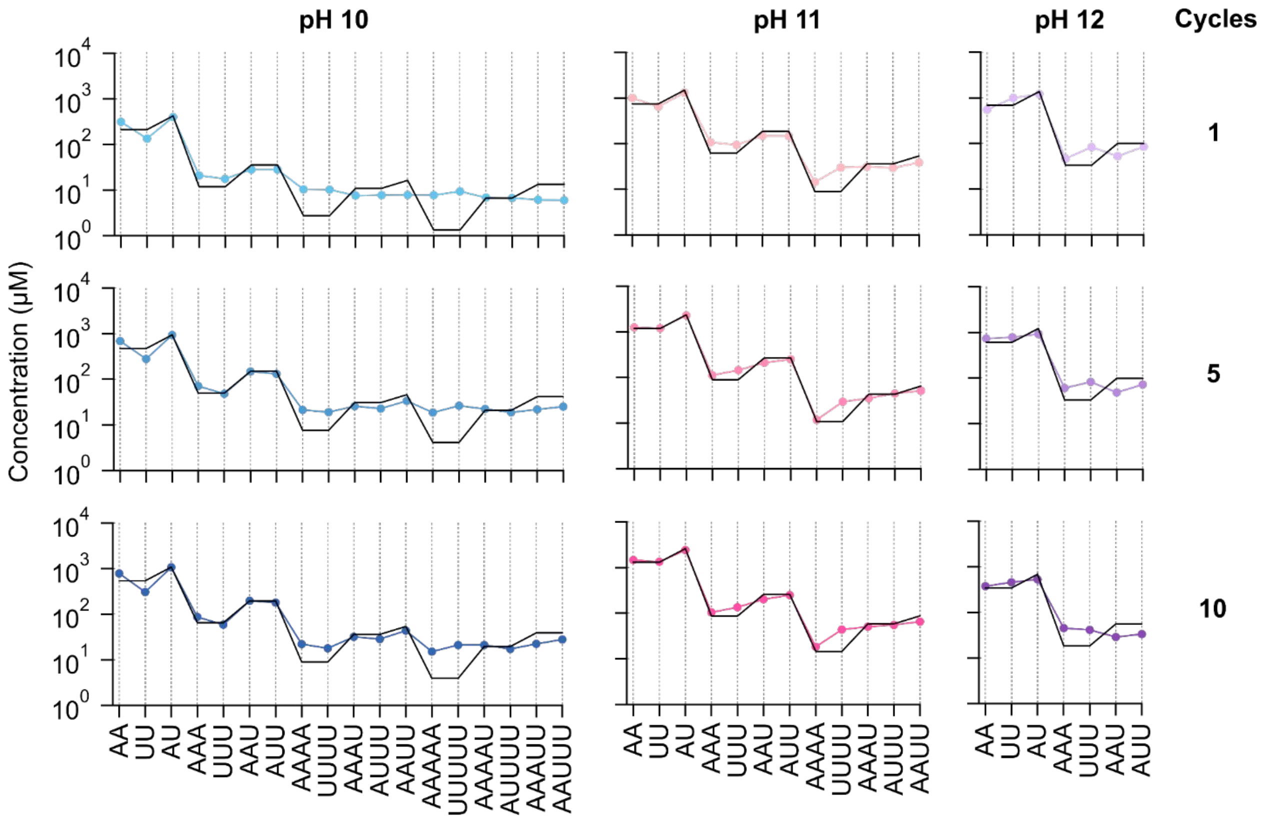

Figure S38. Comparison between experimental data for the AU mixed samples and theoretical behavior expected for random polymerization, for pH 10, 11, 12 and the 1st, 5th and 10th cycle. The full black line corresponds to the expected products distribution for a perfectly random polymerization. The colored dots correspond to the experimental data obtained by LC-MS analysis.

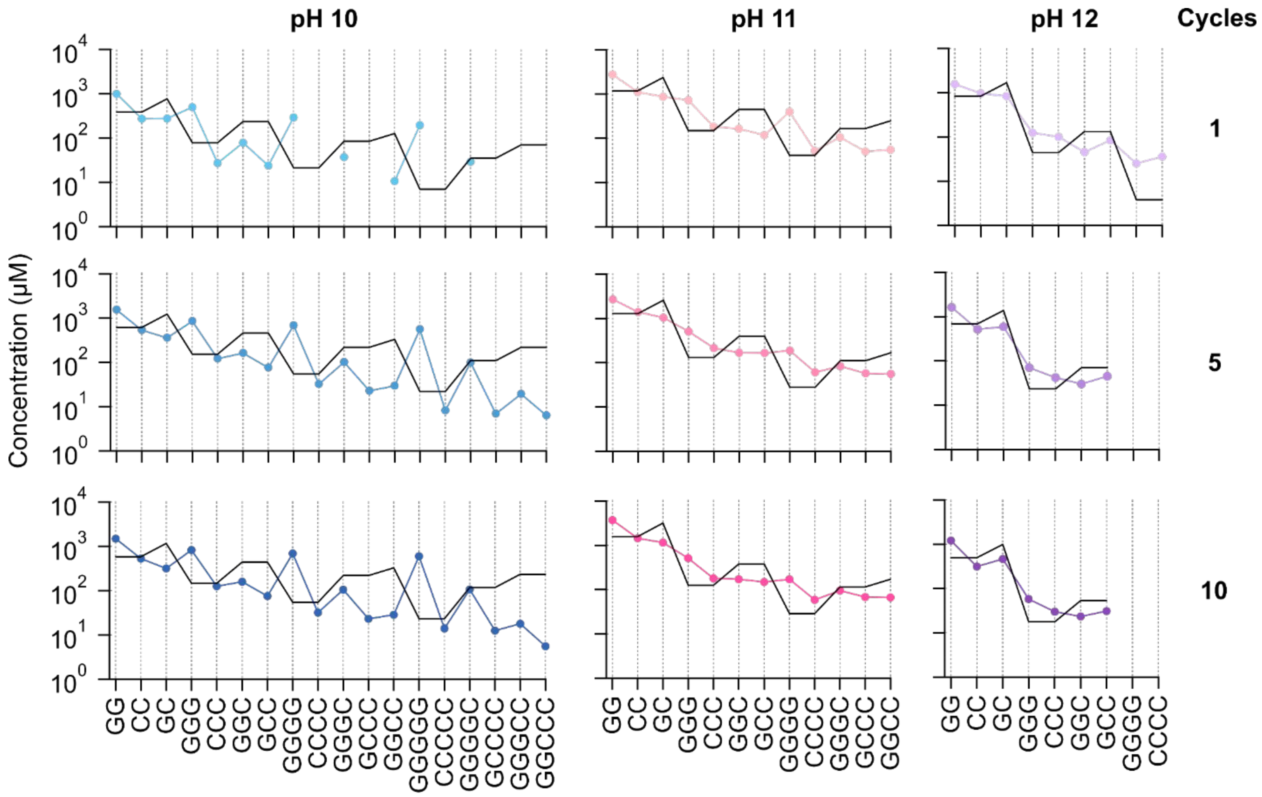

Figure S39. Comparison between experimental data for the GC mixed samples and theoretical behavior expected for random polymerization, for pH 10, 11, 12 and the 1st, 5th and 10th cycle. The full black line corresponds to the expected products distribution for a perfectly random polymerization. The colored dots correspond to the experimental data obtained by LC-MS analysis.

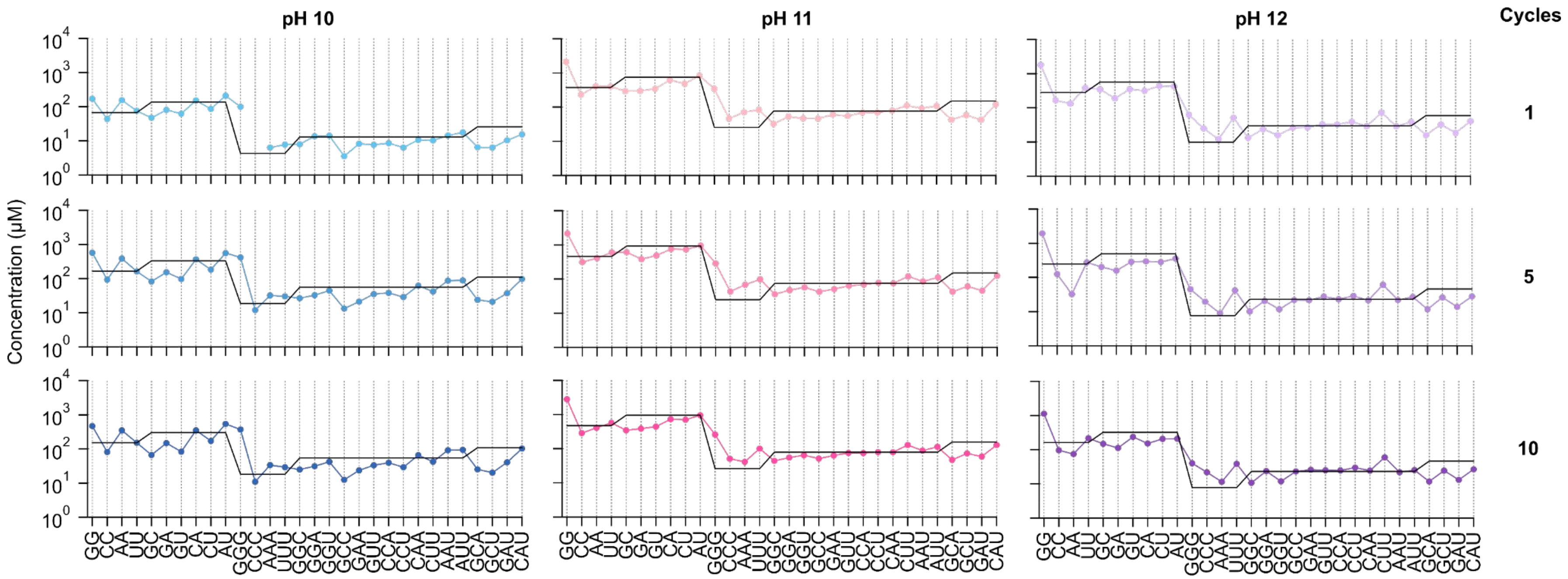

Figure S40. Comparison between experimental data for the AUGC mixed samples and the theoretical behavior expected for random polymerization, for pH10, 11, 12 and the 1st, 5th and 10th cycle. The full black line corresponds to the expected products distribution for a perfectly random polymerization. The colored dots correspond to the experimental data obtained by LC-MS analysis.

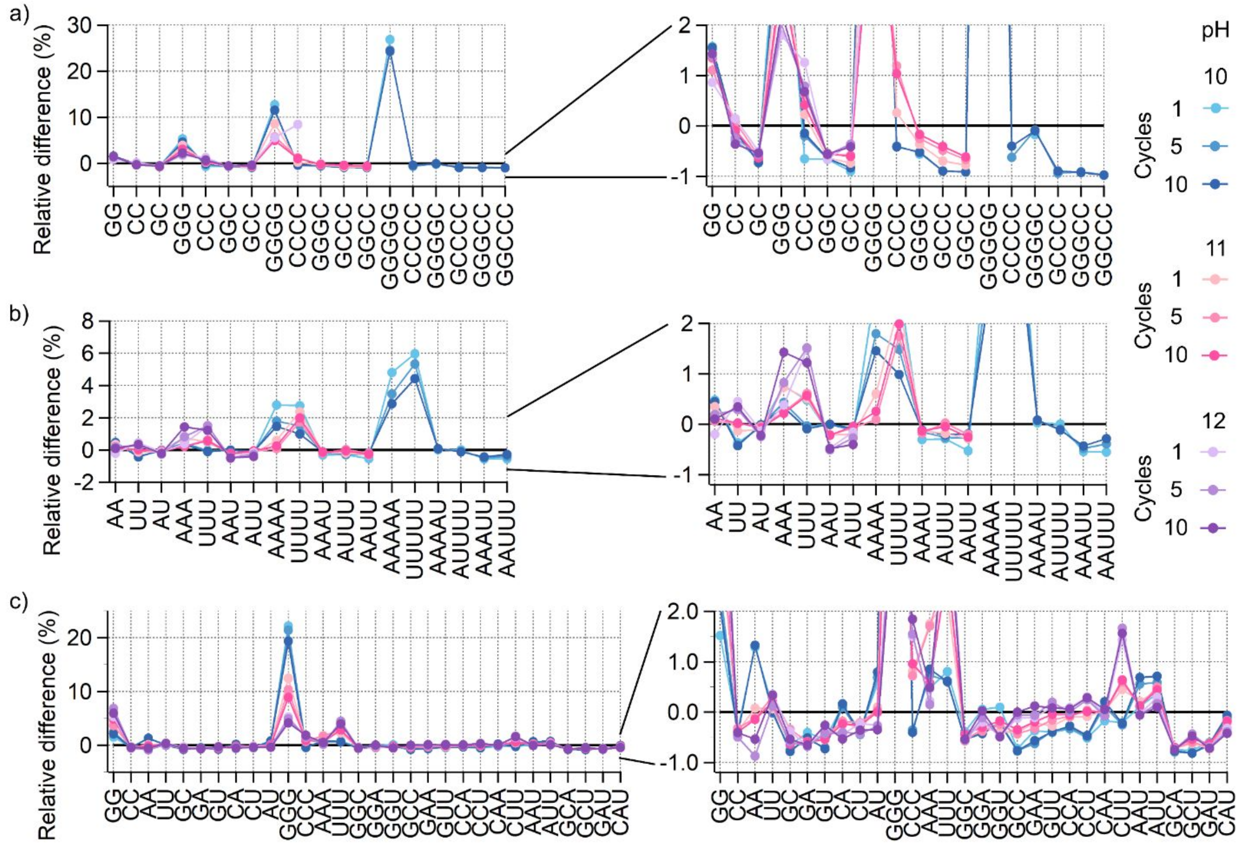

Figure S41. Relative difference between experiment and theory for a) GC mixed samples (for the range [-5 ; 30] and [-1 ; 2]), b) AU mixed samples (for the range [-2 ; 8] and [-1.2 ; 2]), c) AUGC mixed samples (for the range [-5 ; 25] and [-1.2 ; 2]), and for pH = 10, 11, 12 and the 1st, 5th and 10th cycle. For each oligomer sequence, the relative difference corresponds to the difference between experimental and expected concentration and divided by the expected concentration. At increasing pH and cycle number the relative difference for the homo-polymers is found to generally decrease and that of the hetero-polymers, which is negative, is found to increase, indicating a more even inclusion of the different nucleobases in the produced oligomers.

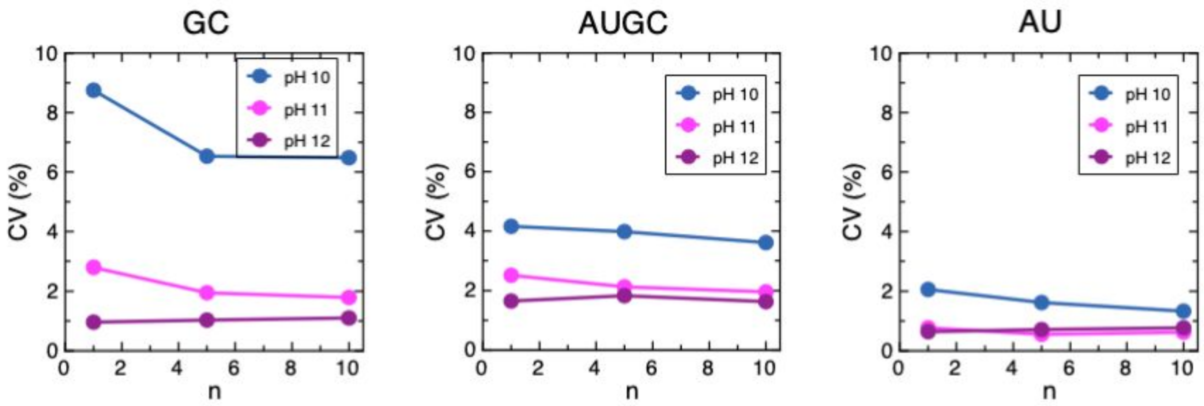

Fig S42. Coefficient of variation of the sequence distributions with respect to the random polymerization for GC, AUGC and AU mixtures. The coefficient of variation, σ, (or the relative standard deviation) distribution has been calculated by the equation:

$$\sigma = \sqrt{\frac{1}{N}\sum_{Ni=1}^{N}\left(\frac{c_{Ni}}{ct_{Ni}} - 1\right)^2}$$

Where c_{Ni} is the measured concentration of the sequence *Ni* (*Ni* = AA, UU, AU, AAA, UUU, AUU, AAU, etc.), ct_{Ni} its expected concentration for random polymerization and N is the total number of different sequences analyzed (data from **Fig. S38-40**). The values of σ are found to generally decrease at increasing pH and cycle number, indicating that the measured sequence distribution is approaching the expected one for perfectly random polymerization, i.e. even inclusion of each nucleobase in the produced oligomers.

Supporting references

1. Dass, A.V.; Wunnava, S.; Langlais, J.; von der Esch, B.; Krusche, M.; Ufer, L.; Chrisam, N.; Dubini, R.C.A.; Gartner, F.; Angerpointner, S.; et al. RNA Oligomerisation without Added Catalyst from 2′,3′-Cyclic Nucleotides by Drying at Air-Water Interfaces**. *ChemSystemsChem* **2023**, *5*, 1–9, doi:10.1002/syst.202200026.

2. Kessner, D.; Chambers, M.; Burke, R.; Agus, D.; Mallick, P. ProteoWizard: Open Source Software for Rapid Proteomics Tools Development. *Bioinformatics* **2008**, *24*, 2534–2536, doi:10.1093/bioinformatics/btn323.

3. Davidson, Z.S.; Huang, Y.; Gross, A.; Martinez, A.; Still, T.; Zhou, C.; Collings, P.J.; Kamien, R.D.; Yodh, A.G. Deposition and Drying Dynamics of Liquid Crystal Droplets. *Nat. Commun.* **2017**, *8*, 1–7, doi:10.1038/ncomms15642.
